# A hydrated 2,3-diaminophenazinium chloride as a promising building block against SARS-CoV-2

**DOI:** 10.1038/s41598-021-02280-5

**Published:** 2021-11-30

**Authors:** Rajani Kanta Mahato, Ayan Kumar Mahanty, Muddukrishnaiah Kotakonda, Sunnapu Prasad, Subires Bhattacharyya, Bhaskar Biswas

**Affiliations:** 1 Department of Chemistry, University of North Bengal, Darjeeling, 734013 India; 2 Department of Biotechnology, University of North Bengal, Darjeeling, 734013 India; 3 Department of Technology, Anna University, Chennai, 600025 India; 4 Department of Pharmaceutical Chemistry, Sri Ramakrishna Institute of Paramedical Science, Coimbatore, 641044 India; 5 Vice-Chancellor, University of North Bengal, Darjeeling, 734013 India

**Keywords:** Biotechnology, Drug discovery, Microbiology, Chemistry, Computational models

## Abstract

Phenazine scaffolds are the versatile secondary metabolites of bacterial origin. It functions in the biological control of plant pathogens and contributes to the producing strains ecological fitness and pathogenicity. In the light of the excellent therapeutic properties of phenazine, we have synthesized a hydrated 2,3-diaminophenazinium chloride (DAPH^+^Cl^−^·3H_2_O) through direct catalytic oxidation of *o*-phenylenediamine with an iron(III) complex, [Fe(1,10-phenanthroline)_2_Cl_2_]NO_3_ in ethanol under aerobic condition. The crystal structure, molecular complexity and supramolecular aspects of DAPH^+^Cl^−^ were confirmed and elucidated with different spectroscopic methods and single crystal X-ray structural analysis. Crystal engineering study on DAPH^+^Cl^−^ exhibits a fascinating formation of (H_2_O)_2_…Cl^−^…(H_2_O) cluster and energy framework analysis of defines the role of chloride ions in the stabilization of DAPH^+^Cl^−^. The bactericidal efficiency of the compound has been testified against few clinical bacteria like *Streptococcus pneumoniae, Escherichia coli*, *K.* *pneumoniae* using the disc diffusion method and the results of high inhibition zone suggest its excellent antibacterial properties. The phenazinium chloride exhibits a significant percentage of cell viability and a considerable inhibition property against SARS-CoV-2 at non-cytotoxic concentration compared to remdesivir. Molecular docking studies estimate a good binding propensity of DAPH^+^Cl^−^ with non-structural proteins (nsp2 and nsp7-nsp-8) and the main protease (M^pro^) of SARS-CoV-2. The molecular dynamics simulation studies attribute the conformationally stable structures of the DAPH^+^Cl^−^ bound M^pro^ and nsp2, nsp7-nsp8 complexes as evident from the considerable binding energy values, − 19.2 ± 0.3, − 25.7 ± 0.1, and − 24.5 ± 0.7 kcal/mol, respectively.

## Introduction

**Figure 1:**
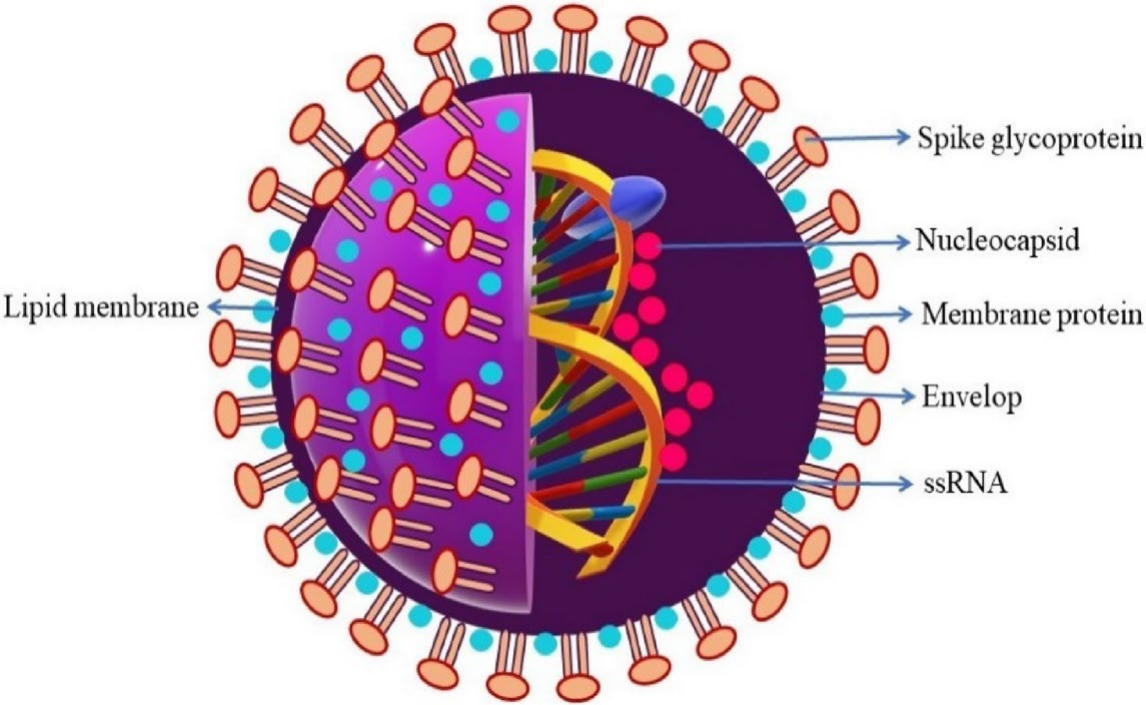
Schematic diagram of SARS-CoV-2.

The relentless outbreak of the novel coronavirus infection caused by severe acute respiratory syndrome coronavirus 2 (SARS-CoV-2) shatters human life and health to a severe extent throughout the world^1^. The infection caused by the novel coronaviruses is not only limited to humans and mammals, but birds are also a sufferer of this disease. Among the identified viral species, six coronavirus species are SARS-CoV, MERS-CoV, HCoV-229E, HCoV-NL63, HCoV-OC43 and HCoV-HKU1 induce severe damage to the living body^2,3^. It is documented that in late 2019, a viral respiratory illness was caused and rapidly spread by SARS-CoV-2 as named by the International Committee of Taxonomy of Viruses (ICTV)^4^. The 2019-nCoV virus is believed to belong to the genus beta-coronavirus of the family Coronaviridae. The 2019-nCoV adopts a single strand, positive-sense ribonucleic acid (RNA) genome and is thought to be originated from bats (Fig. 1)^5–7^. The clinical manifestations of COVID-19 suggest that the disease spreads through respiratory droplets of the infected individuals and leads to life-threatening conditions, including invasive lesions in the lungs leading to a serious threat to human civilizations and the living world^8–12^. On 12 March 2020, the World Health Organization (WHO) declared the COVID-19 a pandemic^13^.

More surprisingly, the disease in the second pandemic wave displays more aggravation leading to acute respiratory failure, sepsis and death^14–17^. The multiple factors like an exacerbated inflammatory reaction, importunate viral load and flawed antiviral defense pathways are responsible^14–18^. Therefore, exploring the underlying cellular mechanisms is of supreme significance to shed off the COVID-19 physiopathology and develop appropriate therapies. The considerable efforts made in scientific research during the last 2 years in developing therapeutic agents and treatment processes are genuinely praiseworthy^19–25^. However, the vast population, lack of awareness, and limited medicinal resources (medicines, vaccines, and medical staff) impose various restrictions to recover this pandemic. Therefore, valuable inputs from the scientific community may advance medical science to a greater extent.

**Figure 2:**
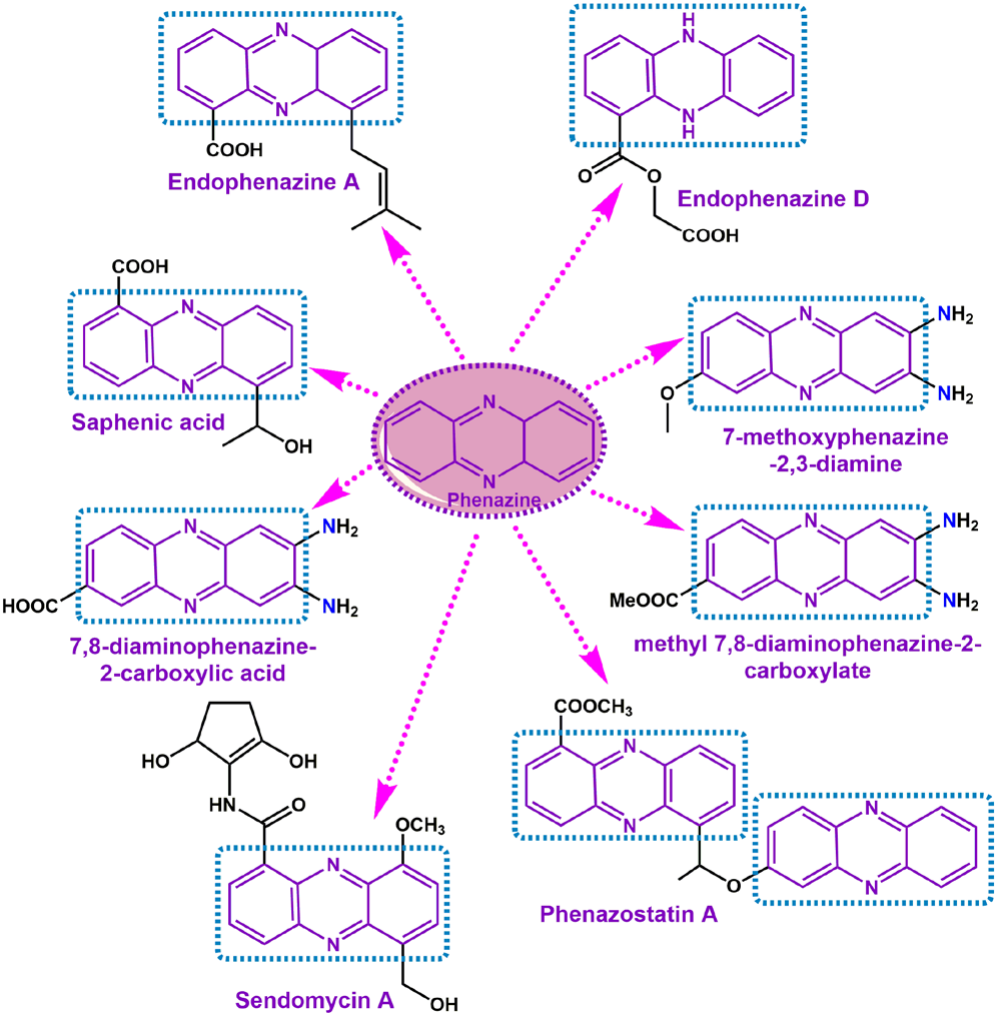
Representative examples of drugs comprising phenazine pharmacophore.

Scientific literature suggests that phenazine is an important class of natural products found in nature. It has excellent medicinal importance. More than 6000 phenazine-based compounds are reported in the last century, and the number is increasing day by day for its clinical and therapeutical efficacy (Fig. 2). Several naturally found phenazines are isolated from gram-positive and gram-negative pathogens, from soil habitants and marine habitats^26,27^. Phenazines are commonly important for their antibiotic^28^, antitumor^29^, antiparasitic^30^ and anti-malarial^31^ properties. Besides that, this redox-active nitrogen-containing heterocyclic pigment balances the redox activities inside their producers^32,33^.

Under the gravity of rapid evolution and the uncontrollable expansion of SARS-CoV-2, we have synthesized a novel hydrated phenazinium chloride salt through a catalytic oxidative coupling of OPD in a pure single crystalline phase. The compound has been characterized with a suite of spectroscopic methods and single crystal X-ray diffraction study. Further, the bio-potency of the compound has been evaluated through in vitro bactericidal and in vitro antiviral studies against few bacterial species and 1 × 10e4VeroE6 cells, respectively. Detailed molecular docking and MD simulation studies are also carried out in triplicate to reveal the binding propensities and stability of the hydrated phenazine chloride bound M^pro^, nsp2 and nsp7-nsp8 complexes.

## Methods and modeling

### Preparation of the 2,3-diaminophenazinium chloride (DAPH^+^Cl^−^)

#### Chemicals, solvents and starting materials

High purity o-phenylenediamine (TCI, Japan), ferric chloride (SRL, India), o-phenanthroline (Merck, India) and ammonium ceric(IV) nitrate and other reagents were purchased from respective outlets. All the used chemicals and reagents were of analytical grade.

#### Synthesis of hydrated DAPH^+^Cl^−^

A previously reported mononuclear iron(III) complex^34^ was used as a catalyst to prepare 2,3-diaminophenazine (DAP) in high yield. o-phenylenediamine (1.08 g, 10 mmol) was added to iron(III) complex (0.55 g, mmol) in a 10:1 mol ratio in ethanol followed by passing of 300 air bubbles with a syringe, and after that, the mixture was kept for slow stirring for 10 h. The pale yellow crystallized product was extracted from the reaction mixture. The compound was recrystallized in an aqueous-ethanol medium. The suitable single crystals were dried in a vacuum over a silica gel indicator.

Isolated yield = 0.910 g (84.2%). Anal. Calc. for C_12_H_17_N_4_O_3_Cl (DAPH^+^Cl^−^): C, 47.92; H, 5.70; N, 18.62; Found: C, 47.97; H, 5.76; N, 18.69. IR (KBr, cm^−1^; Fig. S1): 3412 (ν_O-H_), 3149 (ν_N-H_), 1624(ν_C=N_); UV–Vis (λ_max_, nm; Fig. S2): 258, 426; ^1^HNMR (DMSO-d_6_): δ7.64–7.97 [6H, Ar–*H*], δ6.95 [4H,N*H*_2_].

#### Physical measurements

FTIR-8400S SHIMADZU spectrometer (Shimadzu, Kyoto, Japan) was employed to record IR spectrum of the DAPH^+^Cl^−^ product ranging from 400 to 3600 cm^–1^. ^1^H and ^13^C NMR spectra of the DAPH^+^Cl^−^ were obtained on a Bruker Advance 400 MHz spectrometer (Bruker, Massachusetts, USA) in CDCl_3_at 298 K. Steady-state absorption and other spectral data were obtained on a JASCO V-730 UV–Vis spectrophotometer (Jasco, Tokyo, Japan). A Perkin Elmer 2400 CHN microanalyzer (Perkin Elmer, Waltham, USA) was used to perform the elemental analysis.

#### Crystal structure determination and refinement

X-ray diffraction data of the hydrated DAPH^+^Cl^−^ were collected using a Rigaku XtaLAB Mini diffractometer equipped with Mercury 375R (2 × 2 bin mode) CCD detector. The data were collected with a graphite monochromated Mo-Kα radiation (λ = 0.71073 Å) at 296 K using ω scans. The data were reduced using CrysAlisPro 1.171.39.7f^35^, and the space group determination was done using Olex2. The structure was resolved by the dual space method using SHELXT-2015^36^ and refined by full-matrix least-squares procedures using the SHELXL-2015^37^ software package through the OLEX2 suite^38^.

#### Hirshfeld surface analysis and energy framework analysis

The intermolecular interactions in the DAPH^+^Cl^−^ has been studied and visualized by Hirshfeld surface (HS) analyses with Crystal Explorer 21.2^39,40^. The crystallographic information file of DAPH^+^Cl^−^ was used as an input file in 21.2 Crystal Explorer (Turner et al., 2017, Mackenzie et al., 2017). Various intermolecular interaction energies were calculated for the energy-framework analysis to get insights about different interaction energies involved in stabilizing crystal structure. The energy-framework analysis is a graphical representation of all the stabilizing energy components, e.g. electronic energy, dispersive energy, coulombic energy, total energy, etc., joined as cylinders of various colours between the centroids of the interacting pairs of molecules. It has been illustrated that the strength and contribution of a definite energy component are proportional to the radius of corresponding cylinders joining the molecules. We set the cylindrical radii to a scale factor of 25 and a cut-off value of 0 kJ/mol to visualize the interaction cylinders properly and identify the predominant interaction energy. A single-point wavefunction using the Hartree–Fock method with a 3-21G basis set at a cluster radius of 3.8 Å was generated around the target molecule, and an energy calculation was performed. Proper disorder modelling was done before performing Hirshfeld Surface analysis, and incomplete fragments are removed for computing energy calculation. The interaction energy is broken down as E_tot_ = *k*_*ele*_*E´*_*ele*_ + k_pol_*E´*_*pol*_ + k_*disp*_*E´*_*disp*_ + *k*_*rep*_*E´*_*rep*_. The *k* values are scale factors for benchmarked energy models, *E´*_*ele*_is the electrostatic energy, *E´*_*pol*_ is the polarisation energy, *E´*_*disp*_ is the dispersion energy, and *E´*_*rep*_ is the repulsive energy.

### Bactericidal studies of the DAPH^+^Cl^−^ compound

#### Clinical microbial cultures and culture media

The antibacterial activity of DAPH^+^Cl^−^ was examined against clinical *Streptococcus pneumoniae, Escherichia coli* and *K. pneumonia.* The testing bacterial cultures (clinical cultures) were obtained from a clinical microbiological laboratory, Coimbatore, Tamil Nadu. Muller-Hinton agar media of Himedia Pvt. Himedia sterile discs and Himedia antibiotics disc (Tetracycline, 30 µg), Bombay, India, were used to prepare the media for studying the microbial test. The antibacterial activity of the DAPH^+^Cl^−^ was evaluated by employing a Himedia zone reader.

#### Inoculums and disc preparation

A 100 µL clinical bacterial species, *Streptococcus pneumoniae, Escherichia coli and K. pneumoniae* were inoculated individually in 5 mL of sterile nutrient broth (NB) media and incubated at 37 °C for 24 h. 200 µL from the stock culture was dispensed into 30 mL of sterile nutrient broth and incubated for 24 h to standardize the bacterial culture to 10^8^ CFU/ml (colony forming units).

200 µg of the DAPH^+^Cl^−^ was dissolved in 100 µL sterile water to prepare the stock solution. 50 µL of diluted sample solution from the stock solution was added to Himedia sterile discs under aseptic conditions. Discs were air-dried thoroughly under aseptic conditions and used for the investigation of antibacterial activity.

#### Disc diffusion method (Kirby–Bauer method)

The antibacterial activity of DAPH^+^Cl^−^ and the standard antibiotic, tetracycline, were evaluated following the disc diffusion method. The standardized inoculums (*Streptococcus pneumoniae, Escherichia coli***,** *and Klebsiella pneumoniae*) were inoculated on the Mueller Hinton agar plates using sterile cotton swabs. The compound DAPH^+^Cl^−^ and tetracycline were added to the discs and placed on agar under aseptic conditions. Agar plates were incubated for 30 min at the refrigerator to diffuse the formulation into the agar, and finally, the plates were incubated at 37˚C for 24 h. Afterwards, the inhibition zone developed by the DAPH^+^Cl^−^ was measured with the Himedia zone reader.

### In vitro SARS-COV-2 antiviral activity of the DAPH^+^Cl^−^

#### Cytotoxicity assay against 1 × 10e4VeroE6 cells

The cytotoxicity assay of the synthetic compound DAPH^+^Cl^−^ was performed in a 96-well plate format in a dose-dependent manner^41^. 1 × 10e4 VeroE6 cells were plated per well and incubated at 37 ºC in a humidified 5% CO_2_ for overnight to develop the monolayer formation. After 24 h, 10 µM remdesivir and three different concentrations of the DAPH^+^Cl^−^ (12 µM, 1.2 µM, 0.60 µM) and DMSO were added, and the plates were incubated for 30 h at 37 °C in a humidified 5% CO_2_ (Vatansever et al., 2021 and Zhou et al., 2021). After removal of the cell supernatant, treated cells were stained with Hoechst 33,342 and Sytox orange dye. The images were taken at 10 × , 16 photos per well, which covered 90% of the well area using ImageXpress Microconfocal molecular devices. Hoechst 33,342 nucleic acid stain is a popular cell-permeant nuclear counterstain that emits blue fluorescence when bound to ds-DNA. It stains all the live and dead cells. Sytox orange dye helps to stain the nucleic acids in cells with compromised membranes. This stain is an indicator of cell death. First, the software counted the total number of cells in the Hoechst image. The Sytox image was counted among Hoechst positive cells to determine the number of positive cells for sytox.

#### Immunofluorescence assay (IFA)

The anti-SARS-CoV-2 assay was carried out in a 96 well plate format in which three wells were used for a sample of three different concentrations as previously described by Vernaite et al. 1 × 10^4^VeroE6 cells were plated per well and incubated at 37 ºC in a humidified 5% CO_2_ for 24 h to form a monolayer. In the next day, 10 µM remdesivir and three different concentrations of the DAPH^+^Cl^−^ (12 µM, 1.2 µM, 0.60 µM) were added to the cells, and the plates were incubated for 30 h at 37 ºC in a humidified 5% CO_2_ (Vatansever et al., 2021 and Zhou et al., 2021). The normal VeroE6 cells without DAPH^+^Cl^−^ were considered as a control, while the remdesivir was used as a standard drug used for SARS-CoV-2 treatment as well as to make a comparison of the efficacy of the synthesized phenazinium chloride with remdesivir. The cells were infected with SARS-CoV-2 at an MOI of 0.1 and incubated at 37 ºC in a humidified 5% CO_2_ for 30 h. After 30 h, the cells were fixed in 4% paraformaldehyde. Afterwards, the cells were permeabilized with 0.3% tween-20 and stained with primary and secondary antibodies. The primary antibody-SARS-CoV2 nucleocapsid was the mouse monoclonal antibody (Catalog Number: 40143-MM05) and the secondary antibody was the anti-mouse alexafluor 568. The Hoechst 33,342 stain was used for staining the nucleus. Images were captured and analyzed using ImageXpress Microconfocal devices. The SARS-CoV2 nucleocapsid (Alexa flour-568) and Hoechst nuclei stain images were captured at 10 × , 16 photos per well, covering 85% of the well area. The nucleocapsid positive cells and total nuclei were counted and compared with the control through MetaXpress software using a multi-wavelength cell scoring module.

#### Molecular docking studies, ADME and molecular property prediction

The rationale behind this study is to throw the light on the binding propensities of the hydrated 2,3-diaminophenazinium chloride with main protease (M^pro^) and non-structural (nsp2 and nsp7-nsp8) proteins of SARS-CoV-2. Before the docking study with 2,3-diaminophenazinium chloride, the CIF files of the receptors were fetched from the protein data bank as M^pro^ (PDB ID: 6LU7), nsp2 (PDB ID: 7MSX) and nsp7-nsp8 (PDB ID: 6YHU). Before performing molecular interaction studies, M^pro^, nsp2 and nsp7-nsp8 receptors were further curated for missing side-chain residues using What If interface (https://swift.cmbi.umcn.nl/servers/html/index.html). Molecular docking studies were performed with Autodock v 4.2.6.

#### Preparation of the ligand and receptors

The receptors and DAPH^+^Cl^−^ were prepared by adding polar hydrogen bonds followed by Kollman charge and Gastegier charges. The binding cavity for the DAPH^+^Cl^−^ docking in M^pro^, nsp2 and nsp7-nsp8 were determined from the predefined co-crystallized X-ray structure from RCSB PDB. The residue positions were calculated within 3 Å space from the co-crystallized ligand. After the cavity selection in each case, the co-crystallized ligands were removed using the Chimera tool (https://www.cgl.ucsf.edu/chimera/) and subsequently, energy was minimized using the steepest descent and conjugate gradient algorithm. Then finally, merging the nonpolar hydrogens, both receptor and target compound were saved in pdbqt format.

#### Creating a simulation box

A grid box was created with parameters X = 68, Y = 58, and Z = 64 Å for 6LU7, X = 56, Y = 78, and Z = 61 for 7MSX and X = 108, Y = 78, and Z = 86 for 6YHU with 0.3 Å spacing. Following the Lamarckian Genetic Algorithm (LGA), docking studies of the protein–ligand complex were performed to achieve the lowest free energy of binding (∆G).

#### Validation of docking parameters

During molecular docking studies, three replicates were performed. The total number of solutions was computed 50 in each case, with population size 500, the number of evaluations 2,500,000, the maximum number of generations 27,000, and the rest the default parameters were allowed. After docking, the RMSD clustering maps were obtained by reclustering commands with a clustering tolerance of 0.25 Å, 0.5 Å and 1 Å, respectively, to get the best cluster with the lowest energy score with a high number of populations. The Ki values were determined from the free energy of binding energy using the present algorithm in Autodock 4.2.6. Lipinski’s “Rule of five” was predicted by theoretical in silico ADME calculations^42^. A web tool of Swiss ADME was used to predict Lipinski’s parameters^43^. Addition information related to the details of the molecular docking procedures is furnished provided in supporting information file.

#### Molecular dynamics (MD) simulation and molecular mechanics generalized born surface area (MM/GBSA) calculations

The MD simulations and MM-GBSA calculations were performed for 100 ns in triplicate to reveal the nature of interactions between DAPH^+^Cl^−^ and main protease, M^pro^ (PDB ID: 6LU7), non-structural proteins nsp2 (PDB ID: 7MSX), and nsp7-nsp8 (PDB ID: 6YHU) using the Desmond 2020.1 from Schrödinger, LLC. The OPLS-2005 force field^44–46^ and explicit solvent model with the SPC water molecules were used in this system^47^. Na^+^ ions were added to neutralize the charge. 0.15 M, NaCl solutions were added to the system to simulate the physiological environment. The NPT ensemble was set up using the Nose–Hoover chain coupling scheme^48^ with temperature 27 °C, the relaxation time of 1.0 ps and pressure 1 bar maintained in all the simulations. A time step of 2 fs was used. The Martyna-Tuckerman–Klein chain coupling scheme^49^ barostat method was used for pressure control with a relaxation time of 2 ps. The particle mesh Ewald method^50^ was used for calculating long-range electrostatic interactions, and the radius for the coulomb interactions was fixed at 9 Å. RESPA integrator was used to calculate the non-bonded forces. The root mean square deviation (RMSD) was employed to monitor the stability of the MD simulations.

The binding free energy (∆G_bind_) of the protein–ligand complexes during MD simulation of the proteins with the DAPH^+^Cl^−^ was estimated using MM/GBSA module at the (Schrodinger suite, LLC, New York, NY, 2017-4). The OPLS 2005 force field, VSGB solvent model, and rotamer search algorithms were used to define the binding free energy during the calculation (Wang et al., 2018). The MD trajectories frames were selected at each 10 ns interval after MD run. The following formula was used to calculate the total free energy binding:

1 $$\Delta {\text{G}}_{{{\text{bind}}}} = {\text{ G}}_{{{\text{complex}}}} {-} \, ({\text{G}}_{{{\text{protein}}}} + {\text{ G}}_{{{\text{ligand}}}} )$$

where ∆G_bind_ = binding free energy, G_complex_ = free energy of the complex, G_protein_ = free energy of the target protein, and G_ligand_ = free energy of the ligand. The MMGBSA outcome trajectories were analyzed further for post dynamics structure modifications. Further, for all the 1000 frames of 100 ns MD simulations, the role of non-covalent interactions were estimated in MM/GBSA and plotted in 3D contour. Addition information related to the details of the methods of MD simulations is furnished in supporting information file.

## Results and discussion

### Synthesis and spectroscopic characteristics of DAPH^+^Cl^−^

**Figure 3:**
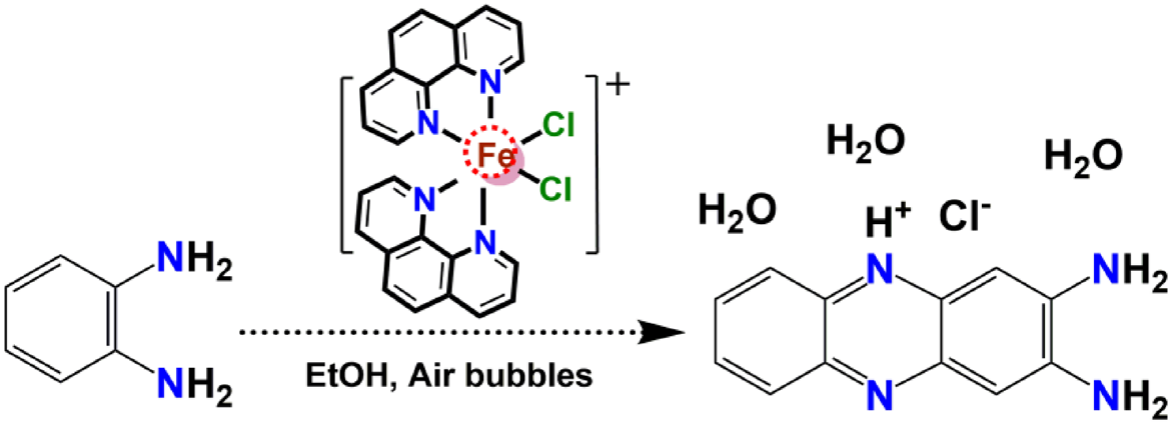
Synthetic route for hydrated diaminophenazinium chloride.

The DAPH^+^Cl^−^ compound was synthesized through a direct catalytic oxidative coupling of o-phenylenediamine with ethanol's previously reported mononuclear iron(III) complex. The reaction was carried out in a 1:10 mol ratio of the iron complex: OPD and purging of 300 air bubbles were required to complete the catalytic oxidation of OPD. The synthetic procedure is shown in Fig. 3.

This structural characteristics and formulation of the DAPH^+^Cl^−^ compound was determined with FT-IR, UV–Vis and ^1^H NMR spectral analysis. FT-IR spectrum of the DAPH^+^Cl^−^ exhibits important characteristics peaks at 3412 (very broad), 3149, 1624 and others (Fig. S1). The characteristic broad band in 3300–3500 cm^−1^ appeared for multiple aqua molecules' O–H stretching frequencies. The important peaks in the region 3300–3350 cm^−1^ and 3184 cm^−1^ attribute to the presence of -NH_2_ groups. It is noticeable that the –NH and –OH stretching frequencies get merged in that region. The characteristic peak at 1624 cm^−1^ is assignable to the azomethine stretching vibration, respectively. These IR spectral data are in well concordance with the previously reported data.^51^ The UV–Vis spectrum of DAPH^+^Cl^−^ in ethanol medium exhibits a high-intensity absorbance band at 258 nm and a moderate intensity electronic transition at 426 nm (Fig. S2). The electronic bands may be corroborated to the π → π* and n → π* electronic transitions of DAPH^+^Cl^−^ (Fig. S2). This observation is in high agreement with the previously reported data of the phenazine produced through ferric chloride and some structurally related compounds^52–54^. The ^1^H NMR spectrum of DAPH^+^Cl^−^ defines the protons' location in DAPH^+^Cl^−^ (Fig. S3). The singlet signals at 11.66 and 10.99 ppm represent the indole-NH protons in DAPH^+^Cl^−^. The methylene protons of DAPH^+^Cl^−^ were also detected and confirmed from the appearance of the signal at 5.82 ppm. The entire protons rise in the region from 8.33 to 6.81 ppm can be assignable to the presence of aromatic protons in DAPH^+^Cl^−^ and agree well with the previously reported data^51,52^.

### Crystal structure, Hirshfeld surface analysis, crystal engineering perspective and energy frameworks

**Figure 4:**
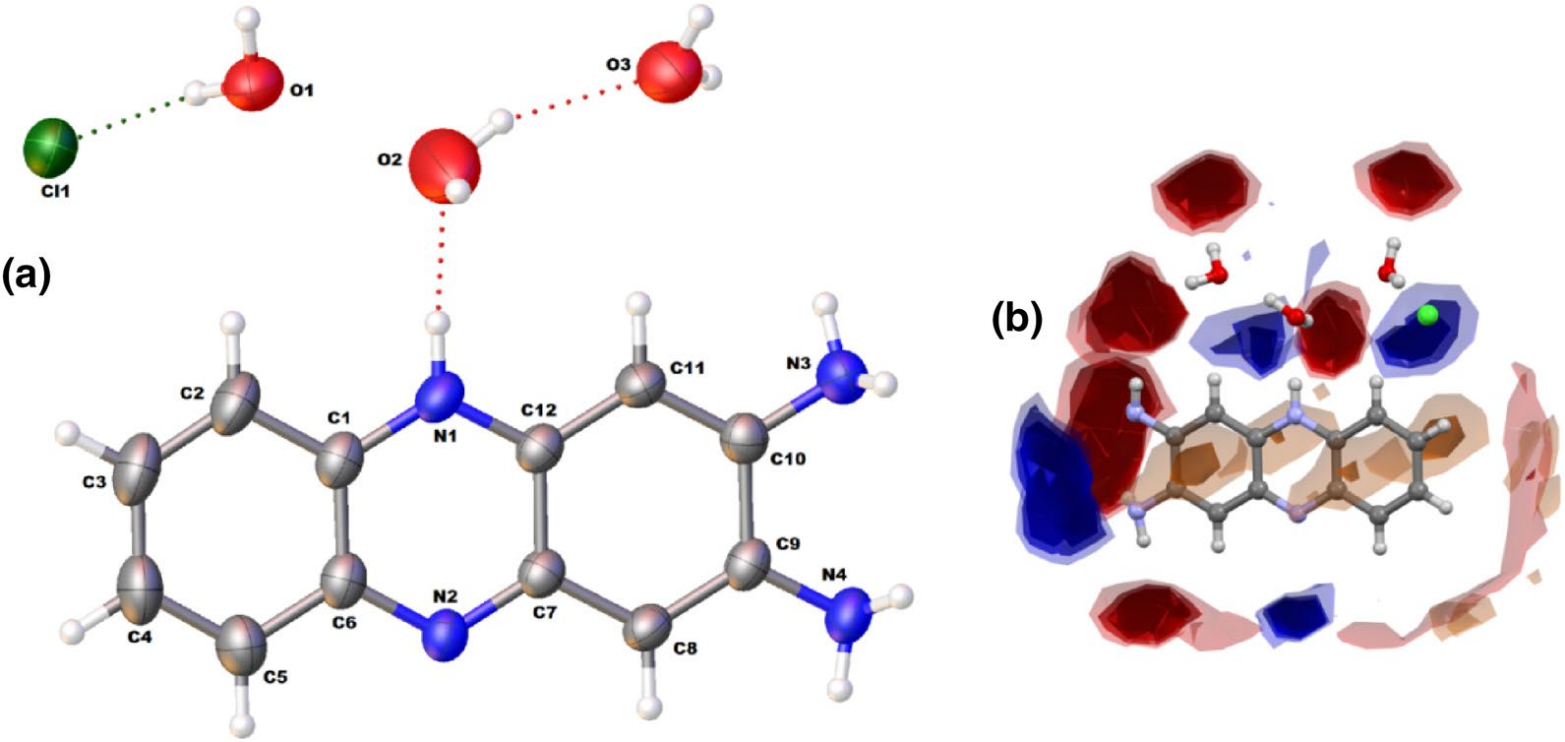
(**a**) An ORTEP diagram of the hydrated DAPH^+^Cl^−^ with 30% probability; (**b**) Interaction map of DAPH^+^Cl^−^ showing the suitability of interactions adopting the intensified red and blue regions.

The single crystals of the oxidation product, 2,3-diaminophenazine (DAP) in its chloride salt, were obtained in catalytic OPD oxidation with the reported iron(III) complex. The crystal structure analysis of DAPH^+^Cl^−^ reveals that the compound crystallizes in a triclinic crystal system with a *P-*1 space group. An ORTEP view of DAPH^+^Cl^−^ is shown in Fig. 4a. The X-ray structure of DAPH^+^Cl^−^ displays an oxidative fusion of two OPD molecules with highly planar aromatic centroids. It is further observed that one of the nitrogen atoms (N1) in the middle aromatic centroid gets protonated, and the cationic charge is counterbalanced with the chloride ion. The crystallographic refining parameters for DAPH^+^Cl^−^ are also given in Table 1. Selected bond distance and bond angles of the compound are shown in Table S1. Further, three water molecules co-exist as solvate molecules with DAPH^+^Cl^−^ and helps the molecular system to get stabilized in the crystalline phase. The generated interaction landscape of DAPH^+^Cl^−^ has been depicted from its 3D coordinates (Fig. 4b).

**Table 1:** Crystallographic data and structure refinement parameters for hydrated DAPH^+^Cl^−^.

Parameters	DAPH^+^Cl^−^
Empirical formula	C_12_H_17_N_4_ClO_3_
Formula weight	300.75
Temperature (K)	296
Crystal system	Monoclinic
Space group	*P*$\overline{1}$
a (Å)	6.8028(11)
b (Å)	9.8872(16)
c (Å)	11.2737(19)
Volume (Å^3^)	713.2(2)
Z	2
ρ (gcm+^3^)	1.401
μ (mm^−1^)	0.281
F (000)	316
R_int_	0.015
θ ranges (°)	2.0–24.7
Number of unique reflections	2897
Total number of reflections	5348
Final R indices	0.0620, 0.1916
Largest peak and hole (eÅ^−3^)	0.51 and − 0.48

The interaction map exhibits the interaction preferences by highlighting regions around the molecule (maps) where chemical functional groups (probes) are likely to contact. Full Interaction maps are instrumental in highlighting the potentiality and understanding the interaction patterns between a ligand and a protein. The red and blue areas in the maps denote the regions in which there is a high probability of locating a hydrogen bond acceptor and H-bond donor, respectively. The brown spots in the map indicate the hydrophobic preferences. Indeed, the observed intensified blue and red landscape around the hydrated DAPH^+^Cl^−^ suggests the worth of attention for its potential candidature against different microbial species (Fig. 4b).

**Figure 5:**
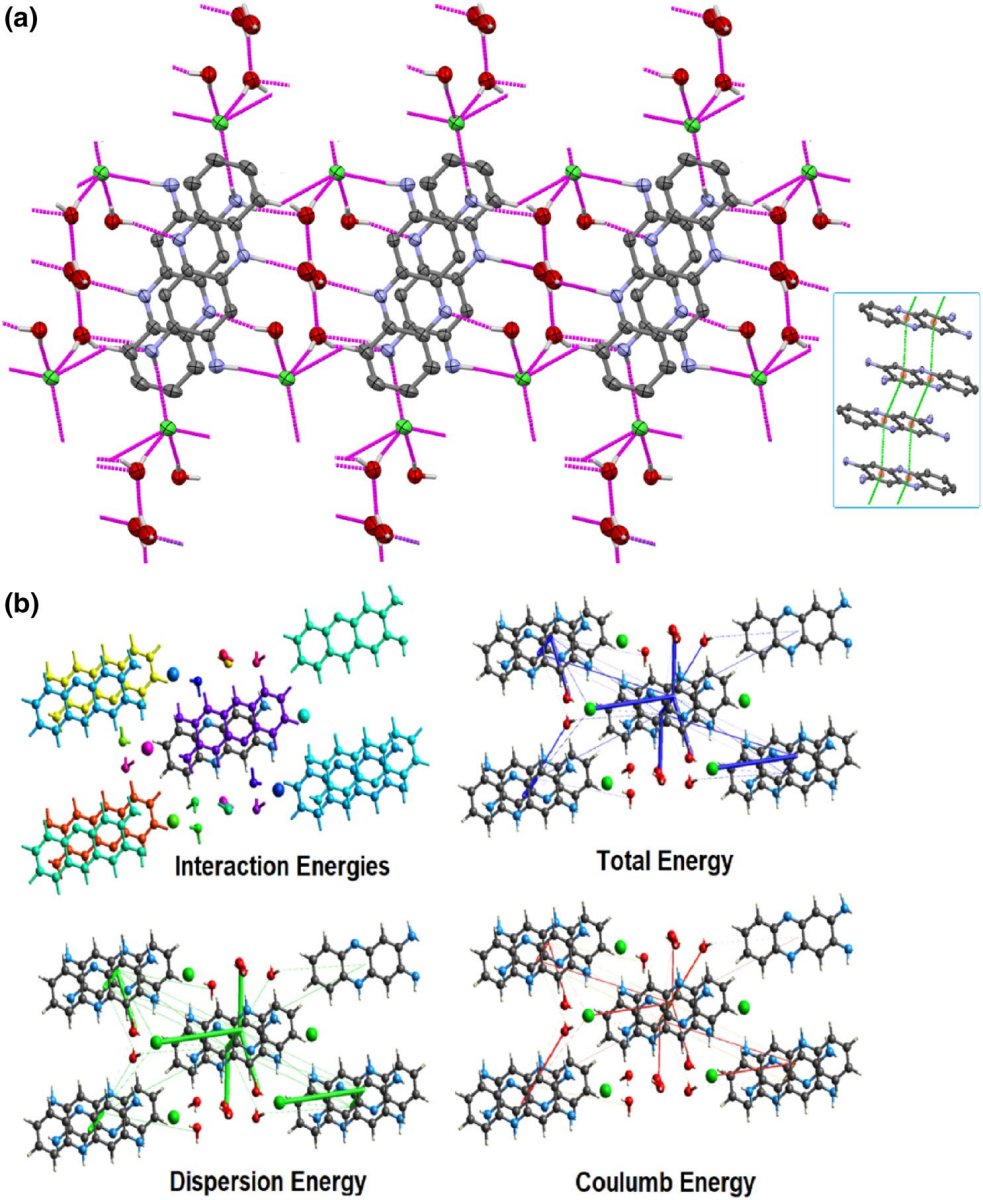
(**a**) Formation of a unique type of (H_2_O)_2_…Cl^−^…(H_2_O) cluster throng strong H…Cl, H…O and H…N interactions and its effect for the construction of supramolecular architecture in DAPH^+^Cl^−^ along *bc* plane; Inset: π…π interactions of AB…AB type (inset); (**b**) Interaction energies participating in the crystal of DAPH^+^Cl^−^ to develop the supramolecular framework.

In the asymmetric unit of DAPH^+^Cl^−^, two of the three crystallized water molecules form strong H-bonding with each other and with the protonated-N of DAPH^+^Cl^−^. Besides this, one crystallizes water interacts with a chloride ion with strong H-bonding in the asymmetric unit. Analysis of the self-assembled architecture for DAPH^+^Cl^−^ suggests that a beautiful (H_2_O)_2_…Cl^−^…(H_2_O) cluster is formed in *bc* plane and displayed in Fig. 5a. This solvent-anion cluster remains an important binder between two cross-linked dimeric DAP units of opposite orientation. The opposite direction of the molecules are further stabilized through strong π…π interactions among the aromatic centroids of dpa and developed AB…AB type of layer in the crystalline phase (Fig. 5a). Notably, the water molecules and chloride ions form an attractive water-chloride cluster. The hydrogen bonding interaction distances were found very strong, starting from 1.89 to 2.38 Ǻ. The intermolecular interaction parameters are given in Table S2.

Moreover, the computational results on the interaction energy frameworks for DAPH^+^Cl^−^ were examined. The interaction energies involved in growing supramolecular framework followed by graphical representation of individual interaction energy components is shown in Fig. 5b. The details of interaction energies are summarized in Table S3. The molecular pair-wise contribution of energies is evaluated, followed by the sum of individual energy components multiplied by scale factor, which furnished the total interaction energy of the crystal. The total interaction energies are electrostatic ($E_{ele}^{\prime }$ =  − 91.3 kJ/mol), polarization ($E_{pol}^{\prime }$ =  − 77.4 kJ/mol), dispersion ($E_{disp}^{\prime }$ =  − 486.4 kJ/mol), repulsion (*E´*_*rep*_ = 264.8 kJ/mol), and total interaction energy (E_tot_) was calculated as − 366.92 kJ/mol (Fig. 5b). Hence, the supramolecular architecture is highly stable with total interaction energy of − 366.92 kJ/mol and dispersive energy interactions dominate the framework.

The Hirshfeld surface analysis was further studied for DAPH^+^Cl^−^ to reveal the binding fate of the chloride ion for the stabilization of DAPH^+^ species. The Hirshfeld surface was examined over a definite d_norm_ (− 0.7308 to 1.1993 a.u.) and a view of Hirshfeld surfaces of DAPH^+^Cl^−^ mapped over d_norm_, shape index, curvedness and fragment patch is shown in Fig. S4. The surface over a definite d_norm_ showed a wide area of multiple red spots, which suggests the presence of strong to very strong H…Cl and H…O interactions (Fig. S4). The surface mapped over shape index ranging − 1.00 to + 1.00 a.u. for DAPH^+^Cl^−^ exhibited intense red and blue spots, ensuring the presence of H…Cl and H…O and weak π…π/C-H…π interactions among the dpa units (Fig. 5a). In addition, 2D fingerprints (Fig. S5) plots were also calculated (Table S4), which displayed the active involvement of the H-bonded and π…π interactions in the crystalline phase.

### *Bactericidal activity of DAPH*^+^*Cl*^−^

The bactericidal activity of the hydrated DAPH^+^Cl^−^ was studied against the clinical bacterial species *Streptococcus Pneumoniae, Escherichia coli* and *K.* *pneumoniae* following a disc diffusion method. The results of the inhibition zone diameters are shown in Fig. S6 and tabulated in Table S5. The minimum inhibitory concentration (MIC) values were estimated for DAPH^+^Cl^−^ and tetracycline under identical experimental conditions against *Streptococcus Pneumoniae* to understand the potency of the antibacterial efficiency. The MIC values were determined as 32.5 µg/mL for DAPH^+^Cl^−^ and 29.0 µg/mL for tetracycline. The comparable MIC value of DAPH^+^Cl^−^ with respect to tetracycline certainly recommends the competent inhibition activities of DAPH^+^Cl^−^ against the growth of bacterial species.

Furthermore, transmission electron microscope images were recorded on the *Streptococcus Pneumoniae* bacterial cells isolated from the MIC. The isolated bacterial cells were sputter-coated with a thin layer of gold and observed under a scanning electron microscope. The TEM images of the DAPH^+^Cl^−^ treated bacterial cells and DAPH^+^Cl^−^ untreated control bacterial cells are shown in Fig. S7. The electron microscope scanning images showed the wrinkling of the bacterial cells and portrayed the destruction of the cell membrane of *Streptococcus Pneumoniae* bacterial.

### In vitro* SARS-COV-2 screening activity of DAPH*^+^*Cl*^−^

#### Cytotoxicity of the DAPH^+^Cl^−^

Cell viability and cell toxicity assays are significant for assessing the cellular responses to a tested compound during its screening activity in a biological experiment. Typically, cell viability assay provides an important readout of healthy cells by measuring the metabolic activity or cell proliferation^55^. Cell viability, which measures the proportion of live and healthy cells within a total cell population, can also be estimated by cell toxicity assay through examining cell growth replication. The cytotoxicity of the DAPH^+^Cl^−^ and remdesivir was evaluated independently against 1 × 10e4VeroE6 cells (n = 3) in a dose-dependent manner. The non-cytotoxic concentration was also determined for DAPH^+^Cl^−^ and remdesivir under a similar experimental condition. It is observed that the compounds exhibit a non-cytotoxic concentration against 1 × 10e4VeroE6 cells up to a dose of 12 µM and 10 µM for DAPH^+^Cl^−^ and remdesivir, respectively.

**Figure 6:**
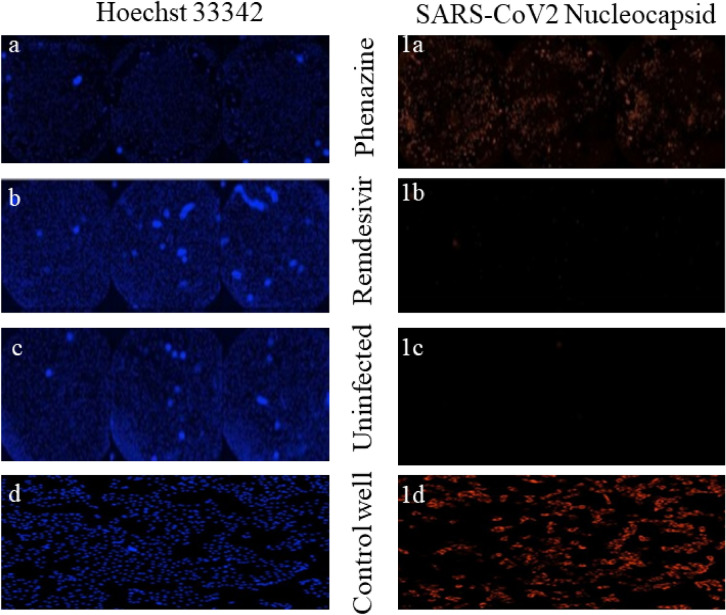
Representative morphological changes were observed in 1 × 10e4VeroE6 cells at the non-cytotoxic concentration of DAPH^+^Cl^−^ and remdesivir using as revealed in Hoechst33342 staining (left) and nucleocapsid staining (right). [**a** to **a1**: VeroE6 cells infected virus treated with DAPH^+^Cl^−^; **b** to **b1**: VeroE6 cells infected virus treated with remdesivir; **c** to **c1**: uninfected VeroE6 cells; d to d1: virus infected VeroE6 cells].

Further, the percentage cell viability of the compounds was also estimated for 1 × 10e4VeroE6 cells (Fig. S8). Compared to the control, the percentage cell viability of the 1 × 10e4VeroE6 cells were determined as 87.3, 97.3, 88.5% at a 0.6, 1.2, 12 µM concentration for DAPH^+^Cl^−^ while 99.23% cell viability was displayed by remdesivir at 10 µM (Table S6). The cytotoxic effect of DAPH^+^Cl^−^ and remdesivir on 1 × 10e4VeroE6 cells is shown in Fig. 6a-d. Therefore, the high percentage of cell viability at a non-cytotoxic concentration of DAPH^+^Cl^−^ against 1 × 10e4VeroE6 cells makes a great promise to develop a potential therapeutic for SARS-CoV-2 under the gravity of present the pandemic.

#### Antiviral efficacy of DAPH^+^Cl^−^ and remdesivir following immunofluorescence assay

The in vitro antiviral activities of the synthetic DAPH^+^Cl^−^ and remdesivir at non-cytotoxic concentrations were further evaluated through immunofluorescence assay (IFA) against VeroE6 cells to understand the viral screening efficacy. It is well documented that remdesivir is a globally prescribed antiviral therapeutic agent for treating SARS-CoV-2, and a comparison of the antiviral activity for DAPH^+^Cl^−^ and remdesivir may put some ray of hope in this context. The anti-SARS-CoV-2 activity was further quantified using primary (mouse monoclonal antibody) and secondary antibodies (anti-mouse alexafluor 568) using IFA (Table S6). 10 µM of remdesivir can significantly inhibit 99.1% of the SARS-CoV-2 infection (Fig. 61b) while DMSO (Dimethyl sulfoxide) as a control didn’t exhibit any inhibition (Fig. 61d). DAPH^+^Cl^−^ at 0.6 µM and 1.2 µM didn’t display any inhibition towards the replication of SARS-CoV-2. However, DAPH^+^Cl^−^ is very effective, as evident from its 70% inhibition activity at 12 µM (Fig. 61a). The uninfected VeroE6 cells are shown in Fig. 61c. Most probably, with the increase of the concentration of DAPH^+^Cl^−^, a cause of substantial interaction with the main protease proteins ceases the replication of RNA genome and result in the prevention of viral attachment to the cells was observed^56^. However, detailed mechanistic research needs to be studied to bring a more scientific vision in this context.

#### Molecular docking studies

**Figure 7:**
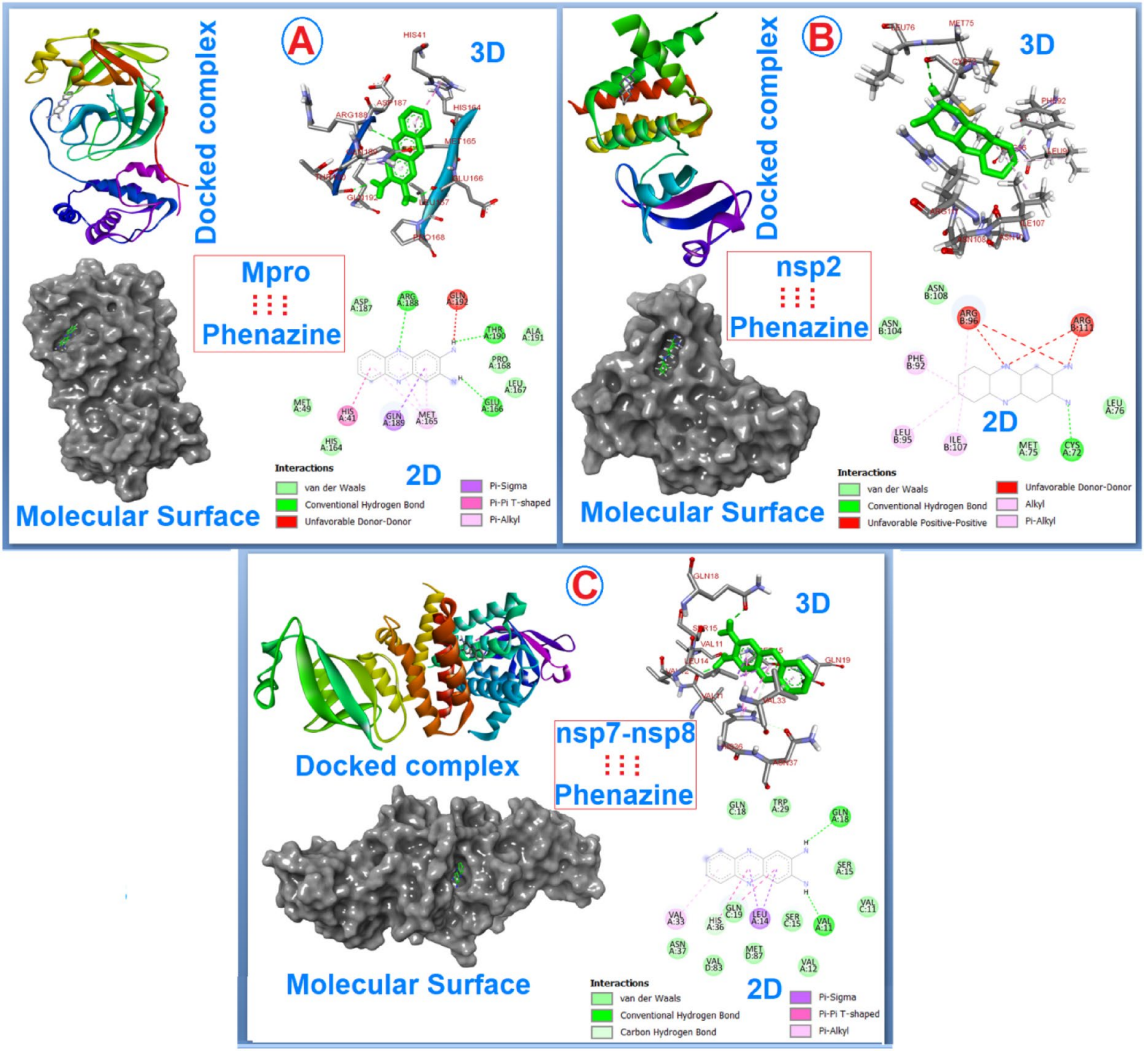
(**A**) Binding motifs of DAPH^+^Cl^−^ with the active sites of M^pro^ along with 2D and 3D modeled interactive plots showing various non-covalent interactions; (**B**) Binding motifs of DAPH^+^Cl^−^ with the binding sites of nsp2 including 2D and 3D modeled interactive plots based on different non-covalent interactions; (**C**) Binding motifs of DAPH^+^Cl^−^ with the binding sites of nsp7-nsp8 with 2D and 3D modeled interactive plots showing various non-covalent interactions.

Molecular docking studies were performed to decipher the binding propensities of DAPH^+^Cl^−^ with the main protease (M^pro^) and non-structural proteins (nsp2 and nsp7-nsp8) of SARS-CoV-2. The images of docked complexes, molecular surfaces, 3D and 2D interactive plots for DAPH^+^Cl^−^ with the proteins of SARS-CoV-2 are shown in Fig. 7. To evaluate the binding interaction between hydrated DAPH^+^Cl^−^ and M^pro^, the best binding pose was obtained from the best RMSD cluster having 0.25 Å tolerances containing 75% population in the same cluster. The DAPH^+^Cl^−^ showed considerable hydrogen bonding interactions with the amino acids Arg188, Thr190 and Glu166 of M^pro^ along with vdw and π…π interactions (Fig. 7A). The effective change of free energy for binding of DAPH^+^Cl^−^ with M^pro^ was estimated as ΔG = –6.66 kcal/mol with predictive inhibition concentration, Ki = 13.11 µM.

The binding interaction of DAPH^+^Cl^−^ with the binding pocket of the non-structural protein, nsp2 showed a substantial binding effect through intermolecular hydrogen bonding, vdw and C…π interactions. Here, the best binding pose was obtained from the best RMSD cluster having 0.25 Å tolerances contain 95% population in the same cluster. The amino acid, Cys72 binds DAPH^+^Cl^−^ with intermolecular hydrogen bonding. However, other non-covalent interactions like vdw forces and C…π interactions were effectively dominant in the binding of DAPH + Cl- with the nsp2 site (Fig. 7B). The change of free energy for binding of DAPH^+^Cl^−^ with nsp2 was noteworthy as ΔG = − 7.91 kcal/mol and Ki = 7.44 µM (Fig. 7B).

Furthermore, the binding effect of DAPH^+^Cl^−^ with nsp7-nps8 was also evaluated and displayed in Fig. 7C. In this binding, the best binding pose was obtained from the best RMSD cluster having 0.25 Å tolerances contain 78% population in the same cluster. It is evident from Fig. 7C that DAPH^+^Cl^−^ compound binds with nsp7-nsp8 through hydrogen bonding, vdw forces, π-sigma and π-alkyl interactions. The change of binding energy of DAPH^+^Cl^−^ with nsp7-nsp-8 was found to be − 7.12 kcal/mol with predictive Ki, 8.61 µM. The details of the interaction between DAPH^+^Cl^−^ and M^pro^, as well as nsp7-nsp8 proteins, are summarized in Table S7.

The molecular docking studies of DAPH^+^Cl^−^ with main protease and non-structural proteins of SARS-CoV-2 suggest that DAPH^+^Cl^−^ displays a good binding propensity with nsp2 protein compared to nsp7-nsp8 and M^pro^ of SARS-CoV-2. The change of free energy for binding of DAPH^+^Cl^−^ with nsp2, nsp7-nsp8 and M^pro^, and Ki values further ensure the predictability for the priority of binding (Table S7). Noteworthy, the structural features of M^pro^, nsp2 and nsp7-nsp8 are entirely different. The predictability of binding through molecular docking does not correlate with the binding priority among the different DAPH^+^Cl^−^ bound protein complexes. However, the negative values for the changes of free energy for binding of DAPH+Cl^−^ with different proteins of SARS-CoV-2 strongly recommend a considerable binding propensity of DAPH^+^Cl^−^ with the proteins. Nevertheless, the tested drug-like nature of DAPH^+^Cl^−^ against SARS-CoV-2 was also proved by calculating ADME values (Table S8). The cytotoxic effect of DAPH^+^Cl^−^ was well recognized as passing Lipinski’s “Rule of 5” with 0 violation which recommends the promising therapeutic behavior against SARS-CoV-2.

The molecular docking results are further corroborated with the earlier reported work^57,58^. Very recently, Hosseini and co-workers reported few potential inhibitors like Ramelteon, Levomefolic acid, Ketoprofen etc. against SARS-CoV-2, which displayed the binding energy ranging between − 6.0 and − 6.66 kcal/mol at the binding cavity of the M^pro^^57^. In contrast, at the same binding cavity, DAPH^+^Cl^−^ displayed to have similar free energy of binding. It is also documented that minimal reports are available for nsp2 inhibition; therefore, the study envisaged here for nsp2 inhibition is a novel addition. Moreover, the inhibition of nsp7-nsp8 by DAPH + Cl^−^ in this study corroborated the same binding site as reported for commercial antiviral drug darunavir with similar range of binding energy (Halder, 2021)^58^.

#### MD simulation and MMGBSA calculations

**Figure 8:**
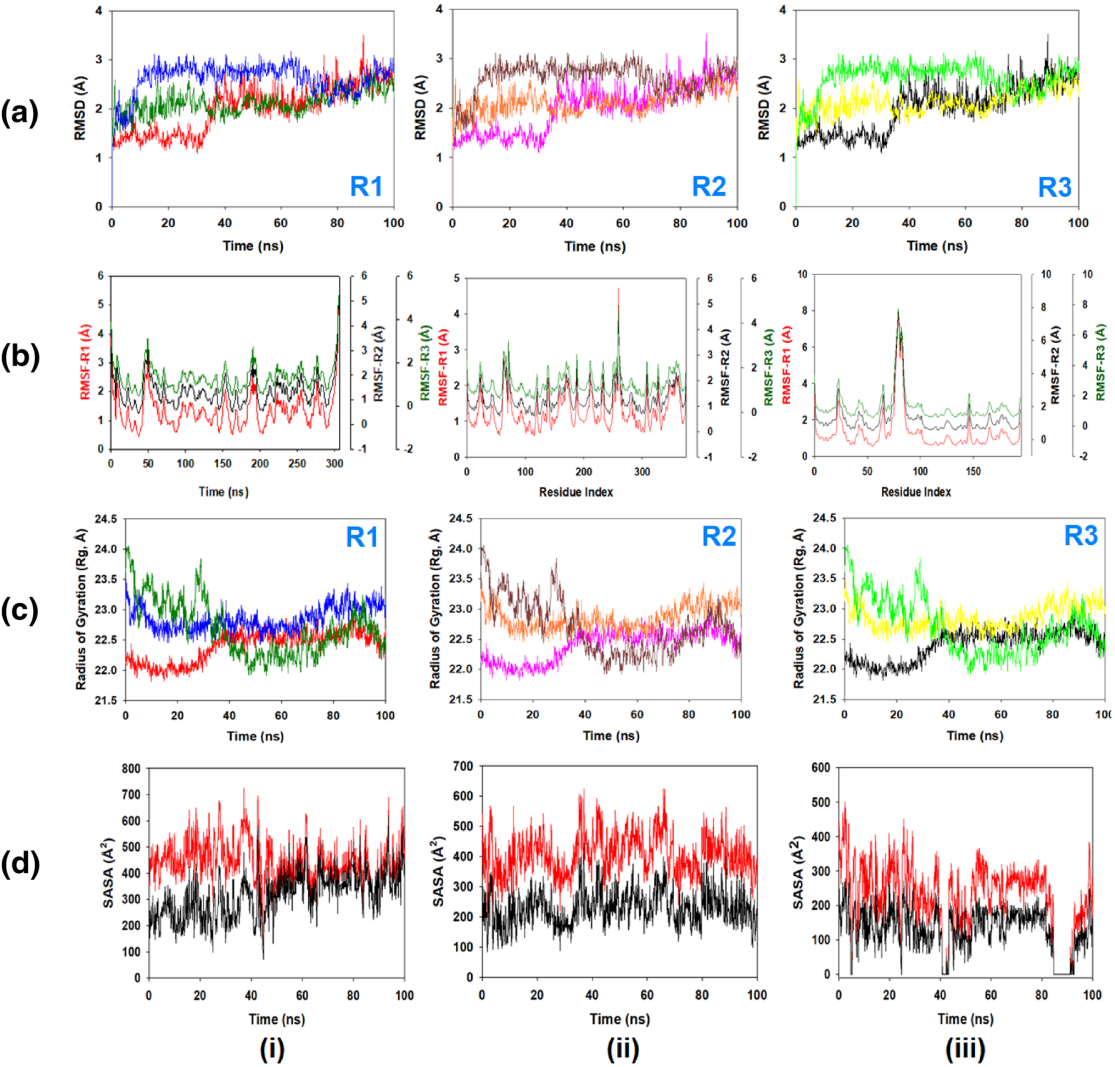
MD simulation trajectory analysis from 100 ns time frame in triplicate displayed (**A**) R1 (replicate 1) RMSD plots of DAPH^+^Cl^−^ bound M^pro^ (red), nsp2 (green) and nsp7-nsp8 (blue), R2 (replicate 2) RMSD plots of DAPH^+^Cl^−^ bound M^pro^ (purple), nsp2 (orange) and nsp7-nsp8 (dark red), R3 (replicate 3) RMSD plots of DAPH^+^Cl^−^ bound M^pro^ (black), nsp2 (yellow) and nsp7-nsp8 (parrot green); (**B**) [Replicate (1, 2, 3), left panel]: RMSF plots of DAPH^+^Cl^−^ bound M^pro^, [replicate (1, 2, 3), middle panel]: RMSF plots of DAPH^+^Cl^−^ bound nsp2, and [replicate (1, 2, 3), right panel]: RMSF plots of DAPH^+^Cl^−^ bound nsp7-nsp8. The RMSF plots of three replicates coded color (red, R1), (black R2) and (green R3). The Y axis scales were adjusted to display the individual replicate plots; (**C**) R1 (replicate 1) Radius of gyration (Rg) plots of DAPH + Cl^−^ bound M^pro^ (red), nsp2 (green) and nsp7-nsp8 (blue), R2 (replicate 2) Radius of gyration (Rg) plots of DAPH^+^Cl^−^ bound M^pro^ (purple), nsp2 (orange) and nsp7-nsp8 (dark red), R3 (replicate 3) Radius of gyration (Rg) plots of DAPH^+^Cl^−^ bound M^pro^ (black), nsp2 (yellow) and nsp7-nsp8 (parrot green); (**D**) Binding SASA in the presence (black) and absence (red) of DAPH^+^Cl^−^ with M^pro^ (left panel), nsp2 (middle panel) and nsp7-nsp8 (right panel). Lowering of SASA (black) signify the binding of ligand to the respective proteins.

Molecular dynamics (MD) simulation of the 2,3-diaminophenazinium bound main protease, (M^pro^) and non-structural proteins (nsp2 and nsp7-nsp8) complexes of the SARS-CoV-2 were studied in detail to understand the nature of possible binding motifs and structurally stable conformations. Replication of the simulations was done in triplicate using the same system parameters to obtain the accurate information of structural convergence in MD studies. The root mean square deviation (RMSD) means a standard measure of structural distance between coordinates. It indicates the extent of structural deviation from its original conformation with time. The RMSD value is a measure of how much the protein conformation has changed with the progress of time. It is well established that increase in the RMSD plot with time, increases the deviation of the protein from its original conformation. The root mean square deviation (RMSD) of Cα-backbone atoms of the 100 ns MD simulation trajectories displayed vibrational deviations with 1.5 Å from beginning to end of the simulation, signifying a stable conformation of the DAPH^+^Cl^−^ bound M^pro^ (Fig. 8A, red). The nsp2 bound complex with DAPH^+^Cl^−^ showed a 0.5 Å displacement of the RMSD (Fig. 8A, green), while nsp7-nsp8 bound DAPH^+^Cl^−^ displayed an overall 0.8 Å deviation (Fig. 8A, blue). In all three replicates, similar displacements were observed (Fig. 8A; R1, R2 and R3). It is evident that nsp2 proteins showed a minimum displacement of the average RMSD (0.5 Å) while the nsp7-nsp8 and M^pro^ displayed a relatively higher degree of average deviation of RMSD. RMSD less than < 1.5 Å for nsp2, nsp7-nsp8 and M^pro^ with respect to the true binding of DAPH^+^Cl^−^ attributing to stable conformations of DAPH^+^Cl^−^ bound complexes and a reasonable estimation of precise calculations of phenazine chloride-proteins interactions.

The root-mean-square fluctuation (RMSF) plot measures the average deviation of a protein residue over time from a reference position (typically the time-averaged position of the protein residue). Thus, RMSF analyzes the portions of structure that are fluctuating from their mean structure the most (or least). It is also evident that lower RMSF values signify the structurally compact conformations while the higher RMSF values indicate more flexible loop regions. RMSF of the amino acid residue position of 100 ns simulation trajectories of DAPH^+^Cl^−^ bound proteins are displayed in Fig. 8B. All the data are measured in triplicates and the Y-axis is repositioned every time to show the individual run outcomes. The individual fluctuations of amino acid residues over a function of time from the reference structure (0 ns) after time 100 ns of the final structure of M^pro^ displayed residue positions 50 and 180 having significant fluctuations averaging 1.5 Å (Fig. 8B, left panel). However, no other important fluctuating residues were observed. While nsp2 bound to DAPH^+^Cl^−^ Cα backbone residues displayed considerable fluctuations at residue positions 70 (1.7 Å) and 260 (2.3 Å) (Fig. 8B, middle panel) and nsp-nsp8 showed at residue position 80 (3.5 Å) (Fig. 8B, right panel). From the average RMSF values of the proteins, it may be suggested that the non-structural proteins exhibited structurally flexible conformations while M^pro^ proteins showed a relatively more compact conformations. Typically, non-structural proteins exist in a more flexible conformation relative to main protease and the results are in well agreement with the natural structural flexibility of the proteins.

The radius of gyration (Rg) is defined as the distribution of atoms of a protein around its axis. The Rg plots were also determined as Rg accounts on the size and compactness of the protein in the ligand-bound state. Lower is the Rg score, higher is the compactness of the docked conformations. The Rg plots are displayed in Fig. 8C. The Rg plot of Cα-backbone indicates that nsp2 protein (Fig. 8C, blue) has a lowering of Rg values from 23.5 to 23.2 Å, meaning compactness with an average of 0.3 Å from the beginning to the end of 100 ns simulation. In contrast, the Rg score in nsp7-nsp8 protein was observed with 1.5 Å total alterations from beginning to end (Fig. 8C, green). However, the Rg plot of M^pro^ displayed deviations from very less significant lowering, thus indicating relatively less compactness of the structure with DAPH^+^Cl^−^ bound state (Fig. 8C, red) as compared to nsp2 and nsp7-nsp8 bound complex.

Followed by Rg analysis, similar patterns were also observed in solvent accessible surface area (SASA) analysis in both ligand-bound and unbound states. It is visible from Fig. 8D that in the unbound state of ligand M^pro^, nsp2 and nsp7-nsp8 displayed high surface area accessible to solvent (Fig. 8D, (i), (ii), (iii), red) while binding with DAPH^+^Cl^−^, the SASA value lowered as compared to the unbound state (Fig. 8D, (i), (ii), (iii), black). This signifies the ligand DAPH^+^Cl^−^ binding compels the respective proteins to become more compact and less flexible. Similarly, Dash and co-workers reported the significance of SASA after ligand binding to the receptor^59^.

**Figure 9:**
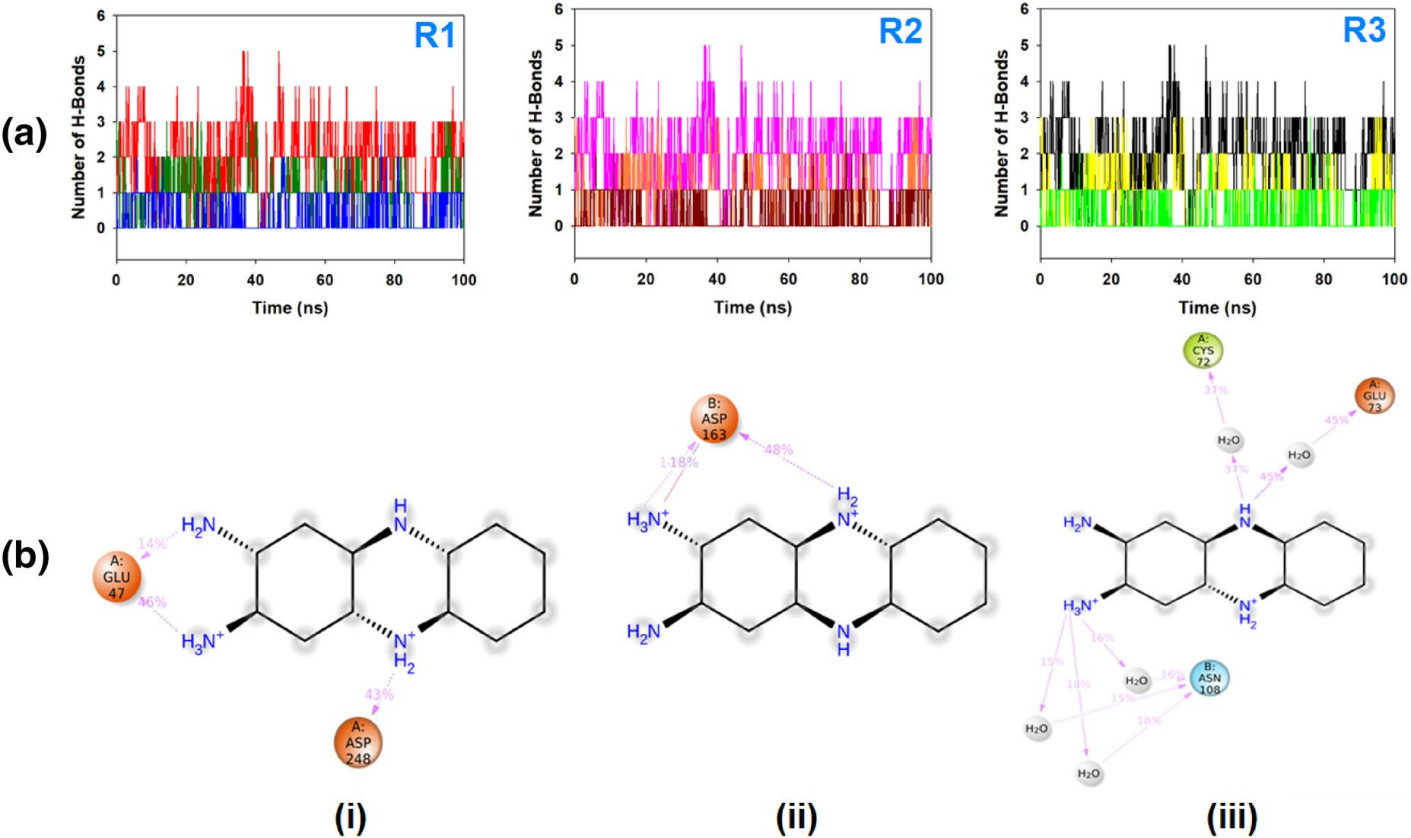
MD simulation trajectory analysis from 100 ns time frame in triplicate displayed (**A**) Number of hydrogen bonds formed between DAPH^+^Cl^−^ and proteins during 100 ns simulation R1 (replicate 1) M^pro^ (red), nsp2 (green) and nsp7-nsp8 (blue); R2 (replicate 2) M^pro^ (purple), nsp2 (orange) and nsp7-nsp8 (dark red) and R3 (replicate 3) M^pro^ (black), nsp2 (yellow) and nsp7-nsp8 (parrot green); (**B**) 2D interaction plot of DAPH^+^Cl^−^ with M^pro^ (left panel), nsp2 (middle panel) and nsp7-nsp8 (right panel) during 100 ns of simulation. Dotted (purple) lines indicate H-bonds, amino acid residues inside sphere and H_2_O displaying water bridges.

The average hydrogen bonds formed between DAPH^+^Cl^−^ and the respective proteins during the 100 ns simulation were also noted and recorded in Fig. 9A. A limited number of hydrogen bonds are displayed in triplicate MD simulations of DAPH^+^Cl^−^ and M^pro^ (Fig. 61A,R1). Overall three hydrogen bonds were formed with Glu47 and Asp248 throughout the simulation and confirmed from 2D ligand binding plot (Fig. 9B, (i)). While, DAPH^+^Cl^−^ bound to nsp2 displayed a couple of hydrogen bonds formed with Asp163 residue throughout the simulation time (Fig. 9A, R2) and also confirmed in 2D interaction plot (Fig. 9B, (ii)). On the other hand, nsp7-nsp8 displayed a noticeable number of hydrogen bond formation with DAPH^+^Cl^−^ (Fig. 9A, R3), and most of the interactions were directed by water bridges as shown in 2D interaction plot (Fig. 9B, (iii)). The existence of hydrogen bonds between proteins and DAPH^+^Cl^−^ has strengthened the binding, helping to make it more stable during the simulation.

**Figure 10:**
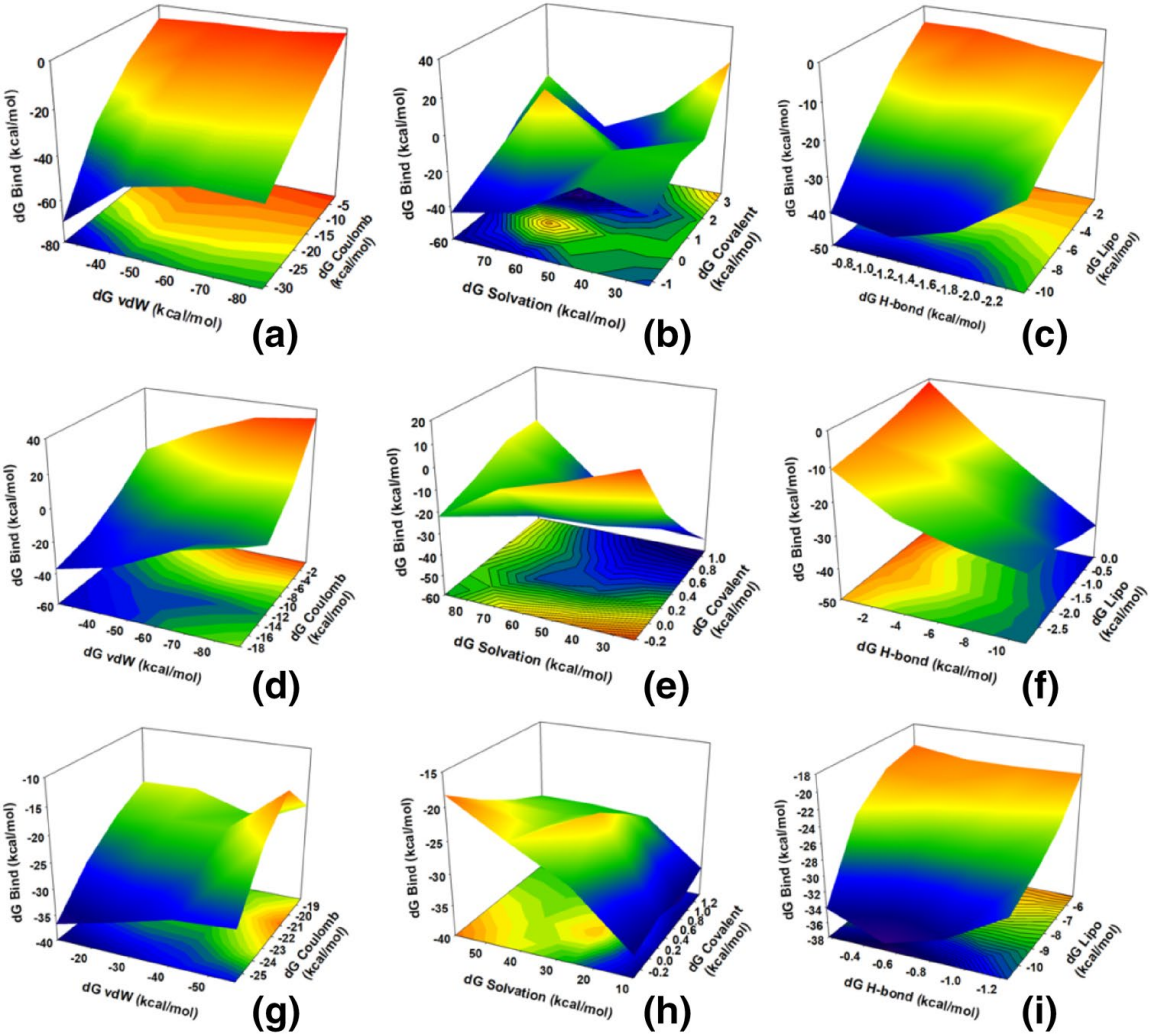
3D contour plots (in sheets) of correlation of non-bonded interactions from MM/GBSA trajectory of 100 ns (all 1000 frames). The principal interacting van der waal’s, coulomb, H-bond, Lipo, salvation and covalent energies are displayed and compared with dG Bind in kcal/mol. (**A**) nsp2 with DAPH^+^Cl^−^ displayed high binding energy due to a good correlation (blue region) of dG vdW and dG Coulomb, (**B**) lessening of binding energy due to negative correlation of dG salvation and dG covalent energies, (**C**) important correlation between dG Lipo and dG H-bond toward augmenting binding energy of nsp2-DAPH^+^Cl^−^ complex; (**D**) nsp-7-nsp8 with DAPH^+^Cl^−^ displayed good binding energy due to considerable correlation (blue region) of dG vdW and dG Coulomb, (**E**) lessening of binding energy due to negative correlation of dG salvation and dG covalent energies, (**F**) important correlation between dG Lipo and dG H-bond toward augmenting binding energy of nsp7-nsp8 DAPH^+^Cl^−^ complex; (**G**) M^pro^ with DAPH^+^Cl^−^ displayed high binding energy due to significant correlation (blue region) of dG vdW and dG Coulomb, (**H**) lessening of binding energy due to negative correlation of dG salvation and dG covalent energies, (**I**) considerable correlation between dG Lipo and dG H-bond toward augmenting binding energy of M^pro^—DAPH^+^Cl^−^ complex. Sheet colour blue indicates the highest correlation, green moderate and yellow least.

Utilizing the 100 ns MD simulation trajectory of the last frame (100 ns), the binding free energy along with other contributing energy in the form of MM/GBSA was determined for DAPH^+^Cl^−^ complexed with M^pro^, nsp2 and nsp7-nsp8. All the triplicate MD simulations were considered and standard deviation is calculated and represented with the binding energies. By considering all the 1000 frames of 100 ns MD simulation, the non-bonded interactions were measured in MM/GBSA and plotted in 3D contour. The binding free energy (dG Bind) of DAPH^+^Cl^−^ with nsp2 displayed –25.7 ± 0.1 kcal/mol. The results displayed in Fig. 10A-C, suggested that the maximum contribution to dG_bind_ in the simulated DAPH^+^Cl^−^ bound nsp2 complex stability through the contribution of dG_bind_ Coulomb, dG_bind_ vdW dG_bind_ H-bond and dG_bind_ Lipo. dG_bind_ Coulomb and dG_bind_ vdW attributed for better correlation in the making toward higher binding energy (Fig. 10A), similarly, dG_bind_ H-bond and dG_bind_ Lipo (Fig. 7C). However, contrasting behaviour showed by dG_bind_ covalent and dG_bind_ salvation energies lowered the binding energy (Fig. 10B). The overall binding free energies are the outcome of all these positive and negatively correlated interacting energies. The binding energy (dG_bind_) of DAPH^+^Cl^−^ bound nsp7-nsp8 was found to be − 24.5 ± 0.7 kcal/mol (Fig. 10D,E,F) and the trajectory analysis for the contribution of total binding free energies displayed a similar pattern of the phenomenon as in the case of nsp2. On the other hand, DAPH^+^Cl^−^ bound M^pro^ displayed dG Bind –19.2 ± 0.3 kcal/mol. The highest binding energies from every 10 ns of M^pro^ trajectories displayed the high contribution of coulombic energy (blue) and van der Wall’s energy toward more negative free energy as (Fig. 10G) and Lipo energy as well as H-bonds energy (Fig. 10I). While solvation energy and covalent energies disturbed the system more toward destabilization with positive free energies (Fig. 10H). While comparing with the binding free energies obtained from docking results, the MM/GBSA energies can be comparable where nsp2, nsp7-nsp8 displayed greater binding as compared to M^pro^ with DAPH^+^Cl^−^.

Therefore, MM/GBSA binding energy analysis from the MD trajectories recommends that the diaminophenazinium salt shows a considerable binding effect of DAPH^+^Cl^−^ with M^pro^, nsp2 and nsp7-nsp8 proteins, leading to stable conformations. Moreover, a positive correlation can be established from the MM/GBSA binding energies and predicted inhibitory concentration (Ki) obtained from the molecular docking studies. As the binding energies increased from M^pro^ to nsp7-nsp8 to nsp2, the Ki values decreased following a similar pattern. Therefore, it can be suggested that the lower the binding energy, low the concentration of DAPH^+^Cl^−^ is required to inhibit the respective proteins. In addition, MD simulation studies and MM/GBSA calculations also attribute a prediction for the better inhibition activity of DAPH^+^Cl^−^ against non-structural nsp2 and nsp-7-nsp8 proteins relative to M^pro^, although the considerable binding effect was observed for each of the proteins.

At present, structural chromophore-based drug design of different synthetic and commercially available compounds grabs a considerable attraction to scientists. Looking at the severe effect of the SARS-CoV-2, many scientists are actively engrossed in searching for potential therapeutics against SARS-CoV-2 (Culletta et al., 2020, Choudhary et al., 2020, Badavath et al., 2020 and references there in)^60–62^. Culletta et al.^60^, explored the inhibition properties of a large number of designed structure-based pharmacophores against the proteins encoded by SARS-CoV-2. They considered 26 experimental drugs, 5 investigational drugs, and 3 approved drugs to study. They carried out molecular docking and MM-GBSA calculations using MD simulations for 100 ns. The drug molecules showed a significant change of dG Bind energy ranging from − 35 to − 90 kcal/mol with the interaction of M^pro^ and nsp proteins. Om Silakari et al.^61^, examined the inhibitory properties of a large number of arbidol analogues (36 molecules) through virtual screening of the proteins of SARS-CoV-2 and reported dG bind energies for the docked complexes through MM-GBSA calculations. Among the studied molecules, A_BR4, A_BR9, A_BR18, A_BR22 were highly interactive with spike proteins and A_BR5, A_BR6, A_BR9, and A_BR18 were effective against the main protease of the SARS-CoV-2. The group further reported the ΔG MM-GBSA energies (kcal/mol) for the main protease docked complexes ranging from − 2 to − 47 kcal/mol. Similarly, Badavath et al.^62^, forecasts a computer-aided drug design for the anti-SARS screening activity of 118 isatin derivatives comprising 16 distinct heterocyclic compounds, 5 natural products and 7 repurposed drugs. The binding propensities of the compounds towards the main protease of SARS-CoV-2 reveal their potential inhibition properties against SARS-CoV-2. Furthermore, Purwati et al.^63^, evaluated the in vitro anti-SARS-COV-2 activity of a series of ratiometrically designed dual combinatory drugs namely Lopinavir–Ritonavir–Clarithromycin, Lopinavir–Ritonavir–Azithromycin, Lopinavir–Ritonavir–Doxycycline, Hydroxychloroquine–Azithromycin, Flaviptravir–Azithromycin against Vero cell lines. The group also determined the cytotoxic concentrations (CC_50_) and IC_50_ values for 24, 48 and 72 h. They reported the CC_50_values in the range 4.2 × 10^2^ to 1.1 × 10^10^ µg/mL and IC_50_ values ranging from 12.1 to 24.90 µM. The in vitro cytotoxicity studies of DAPH^+^Cl^−^ against VeroE6 cell lines showed a non-cytotoxic concentration, 12 µM which enabled 70% inhibition against VeroE6 cell lines.

Nevertheless, on comparison of the binding propensities of the molecules with main protease and non-structural proteins of SARS-CoV-2, it is evident that the synthetic hydrated DAPH^+^Cl^−^ compound shows a good binding effect with main protease (− 19.2 ± 0.3 kcal/mol), nsp2 (− 25.7 ± 0.1 kcal/mol) and nsp7-nsp8 (− 24.5 ± 0.7 kcal/mol) as revealed from MM-GBSA calculations. The changes of binding energies of DAPH^+^Cl^−^ with M^pro^, nsp2 and nsp7-nsp8 proteins are considerable with respect to the reported binding energy values of the reported drugs and clinically approved agents. Furthermore, in silico and in vitro cell viability and immunofluorescence assay of DAPH^+^Cl^−^ against Vero cell lines attributes a good estimation for DAPH^+^Cl^−^ to turn out a potential therapeutic agent against SARS-CoV-2.

## Conclusions

To summarize, we synthesized a hydrated phenazinium chloride through straightforward catalytic oxidation of o-phenylenediamine. Interestingly, we are able to isolate the compound in single crystalline phase in high yield. The crystal structure analysis of the compound reveals that phenazinium ion is stabilized by a chloride ion in association with lattice water. The hydrated DAPH^+^Cl^−^ compound turns out to be a good bactericidal agent against few clinical bacteria. The synthetic compound displays a comparable MIC value with respect to a standard antibiotic, tetracycline, and can destroy the bacterial cell membrane. Further, in vitro SARS-CoV-2 screening activities were evaluated against 1 × 10e4VeroE6 cells through cell viability assay and cytotoxic studies. The synthetic phenazinium salt exhibits ~ 90% cell viability and 70% inhibition activity at the non-cytotoxic concentration, 12 µM, which is comparable to the SARS-CoV-2 screening activity of remdesivir at 10 µM. The molecular docking studies predict a relatively higher binding propensity of DAPH^+^Cl^−^ non-structural proteins (nsp2 and nsp7-nsp8) compared to M^pro^. Different non-covalent interactions like hydrogen bonding, vdw interactions and π-sigma interactions are operative to form stable docked complexes. The MD simulation studies in triplicate for 100 ns show a considerable binding energies (dG Bind) of DAPH^+^Cl^−^ with nsp2, nsp7-nsp8 and M^pro^ as − 25.7 ± 0.1, − 24.5 ± 0.7 and − 19.2 ± 0.3 kcal/mol, respectively. The binding energies estimated by molecular docking and MD simulations analysis set a similar trend of stability for docked complexes and attribute the stable conformations of DAPH^+^Cl^−^ docked main protease and non-structural proteins complexes. Finally, we deeply believe that in silico SARS-COV-2 screening activity and in vitro cytotoxicity against VeroE6 cell lines put a new source of light for suitable structural modifications like extension of aromatization or planarity of the phenazine pharmacophore that may certainly enrich the antiviral activity and may turn out to be a potential therapeutics with a great promise.

## Supplementary Information

Supplementary Information 1.

Supplementary Information 2.

## Data Availability

The data are available from the corresponding author upon reasonable request.
