# Supplementary material for: A hydrated 2,3-diaminophenazinium chloride as a promising building block against SARS-CoV-2
Source: Sci Rep. 2021 Nov 30;11:23122. doi: 10.1038/s41598-021-02280-5 (PMC8633378; doi:10.1038/s41598-021-02280-5)
Supplement: Supplementary file 1 — Supplementary Information 1. [file 41598_2021_2280_MOESM1_ESM.doc]

**Supporting Information**

**A hydrated 2,3-diaminophenazinium chloride as a promising building block against SARS-CoV-2**

Rajani Kanto Mahato,^1^ Ayan Kumar Mahanty,^2^ Muddukrishnaiah Kotakonda,^3^ Sunnapu Prasad,^4^ Subires Bhattacharyya,^5,*^ and Bhaskar Biswas^1,*^

***Preparation of protein and ligand for docking study***

The X-ray crystallographic structures of main protease, M^pro^ (PDB ID: 6LU7) and non-structural proteins, nsp2 (PDB ID: 7MSX), and nsp7-nsp8 (PDB ID: 6YHU) of SARS-CoV-2 were retrieved from the Protein Data Bank (PDB) (http://www.pdb.org) database. Graphical User Interface program “Auto Dock Tools” (ADT) from Molecular Graphics Laboratory (MGL) developed by Scripps Research Institute was employed for the preparation of protein for docking study [S1]. Input file of receptor protein for the docking study is created by taking specific china (Chain A) of the protein (6LU7). The binding cavity for the DAPH^+^Cl^-^ docking in M^pro^, nsp2 and nsp7-nsp8 were determined from the predefined co-crystallized X-ray structure from RCSB PDB. The residue positions were calculated within 3 Å space from the co-crystallized ligand. After the cavity selection in each case, the co-crystallized ligands and water molecules were removed from the PDB crystal structures using the Chimera tool (https://www.cgl.ucsf.edu/chimera/) and finally, the receptors’ .pdbqt files were created by the addition of the polar hydrogen atoms and Kollman united atom charges [S2]. For the purposes of the docking procedure, the ligand of this crystal structure were given a partial charge of the atom using the Austin Model 1 semi-empirical method with Bond Charge Correction (AM1-BCC) while the receptor partial charge was calculated by means of a molecular mechanics approach using AMBER ff14SB force field [S3]. The three dimensional (3D) structure of the phenazinium chloride was directly obtained from the CIF after running the CIF in Mercury software of Cambridge Structural Database (CSD) and further optimization of geometry was carried out with the MOPAC 6 package using the semi-empirical AM1 Hamiltonian [S4]. The input .pdbqt file of the ligands was generated using Auto Dock Tools (ADT). As the ligand phenazinium chloride is a non-peptide, therefore, Gasteiger charge was assigned and then non-polar hydrogen was merged.

***Docking study using AutoDock* v 4.2.6**

All molecular docking simulations were carried out in the AutoDock v 4.2.6 developed by Scripps Research institute [S5] and the results of the docking study and the intermolecular interactions between receptors and the ligand molecules were analyzed using BIOVIA Discovery Studio 2020 (DS), version 20.1.0.0 (Dassault Systèmes BIOVIA, Discovery Studio Modeling Environment, Release 2017, San Diego: Dassault Systèmes, 2016) and Edu pymol version 1.7.4.4 [S6]. The three dimensional (3D) affinity (grid) maps and the grid boxes was created with parameters X=68, Y=58, and Z=64 Å for 6LU7, X=56, Y=78, and Z=61 for 7MSX and X=108, Y=78, and Z=86 for 6YHU with a spacing of 0.3 Å generated by AutoGrid auxiliary program for each of the receptors. The blind docking was generated to cover the entire active site of the receptor protein in order to eliminate any biasness arising during the docking simulation [S7]. Lamarckian genetic algorithm and a standard protocol with default setting of other run parameters were used for docking simulation to achieve the lowest free energy of binding (∆G). During molecular docking studies, three replicates were performed. The total number of solutions was computed 50 in each case, with population size 500, the number of evaluations 2500000, the maximum number of generations 27000, and the rest the default parameters were allowed. After docking, the RMSD clustering maps were obtained by reclustering commands with a clustering tolerance of 0.25 Å, 0.5 Å and 1 Å, respectively, to get the best cluster with the lowest energy score with a high number of populations. For each docking experiments, several runs were performed by the program with one predicted binding mode with each run. All the torsions were allowed to rotate. The predicted inhibitory constant has been calculated using the previously reported standardized equations [S8].

***MD simulation studies***

MD simulations were performed with Desmond 2020.1 from Schrödinger, LLC [S9]*.* The inhibitor- SARS-CoV-2 M^pro^, nsp2, and nsp7-nsp8 proteins complex models were separately placed in the orthorhombic box with a buffer distance of 10 Å in order to create a hydration model. TIP3P water model [S10] was used for creation of the hydration model. The selected ligand-protein complexes were first immersed into SPC (simple point charge) water box, extending 10 Å beyond any of the complex’s atoms. Counter ions (6LU7 - 20 sodium and chloride 22, 6YHU - 1 sodium and 7MSX - 11 sodium and 11 chlorides) were added to neutralize charges. The MD was performed in the NPT ensemble at 300 K temperature and 1.63 bar pressure over 100 ns. Simulations were run with the OPLS-3e force field. This counter ions added in the simulation system by calculating the number of ions required to neutralise the hole system before simulation and the calculations were done using Schrodinger maestro system build default parameters. The substrate was parameterised using default settings in Schrodinger maestro having OPLS -3e 2005 force field [S11].

**References**

[S1] R. Huey, G.M. Morris, The Scripps Research Institute, USA (2008), 54–56.

[S2] A .M. Vijesh, A .M. Isloor, S. Telkar, T. Arumoli, H.K. Fun, Molecular docking studies of some new imidazole derivatives for antimicrobial properties, Arab. J. Chem. 6 (2013) 197–204.

[S3] C.W. Murray, D.A. Erlanson, A.L. Hopkins, G.M. Keserü, P.D. Leeson, D.C. Rees, C.H. Reynolds, N.J. Richmond, Validity of Ligand Efficiency Metrics, ACS Med. Chem. Lett. 5 (6) (2014) 616–618.

[S4] K. Ohtawara, H. Teramae, Study on optimization of molecular structure using Hamiltonian algorithm, Chem. Phys. Letters 390 (2004) 84–88.

[S5] O. Trott, A.J. Olson, AutoDock Vina: improving the speed and accuracy of docking with a new scoring function, efficient optimization and multithreading, J. Comp. Chem. 31 (2020) 455–461.

[S6] W.L. DeLano, Pymol: an open-source molecular graphics tool. CCP4 Newsletter on protein crystallography, 40(1) (2002), 82–92.

[S7] G.M. Morris , D.S. Goodsell , R.S. Halliday , R. Huey , W.E. Hart , R.K. Belew , A.J. Ol- son, Automated docking using a Lamarckian genetic algorithm and an empirical binding free energy function, J. Comp. Chem. 19 (14) (1998) 1639–1662.

[S8] A.L. Hopkins, C.R. Groom, A. Alex, Ligand efficiency: a useful metric for lead selection, Drug discov. Today 9 (10) (2004) 430–431.

[S9] Schrödinger Release 2018-2. Desmond Molecular Dynamics System (D. E. Shaw Research, New York, 2018).

[S10] Jorgensen, W. L., Chandrasekhar, J., Madura, J. D., Impey, R. W. & Klein, M. L. Comparison of simple potential functions for simulating liquid water. *J. Chem. Phys.* **79**(2), 926–935 (1983).

[S11] Choudhary MI, Shaikh M, Tul-Wahab A, Ur-Rahman A. In silico identification of potential inhibitors of key SARS-CoV-2 3CL hydrolase (Mpro) via molecular docking, MMGBSA predictive binding energy calculations, and molecular dynamics simulation. *PLoS One*. 2020;15(7): e0235030.

**Figure S1**. FT-IR spectrum of DAPH^+^Cl^-^

**
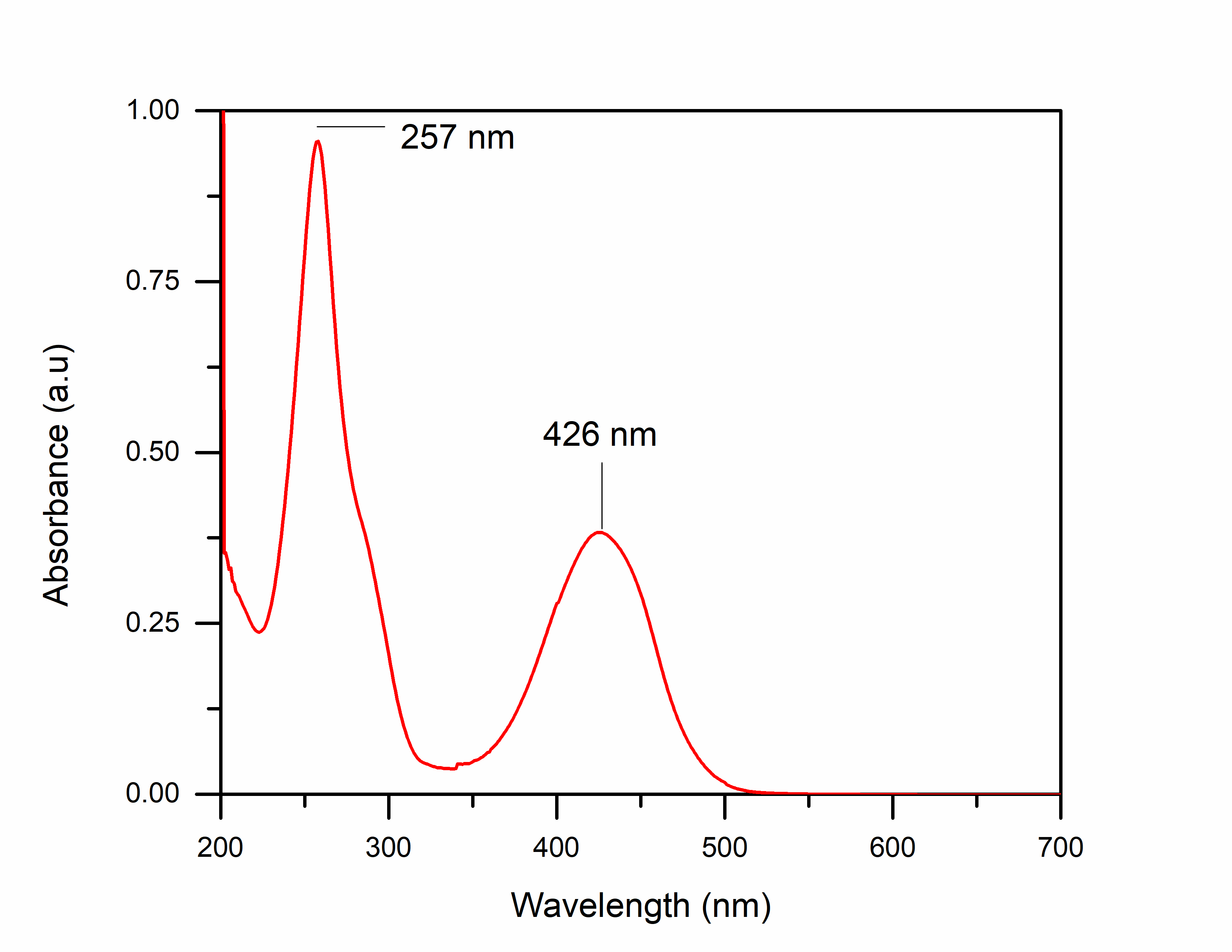
**

**Figure S2.** UV-Vis spectrum of the DAPH^+^Cl^-^ in EtOH

**
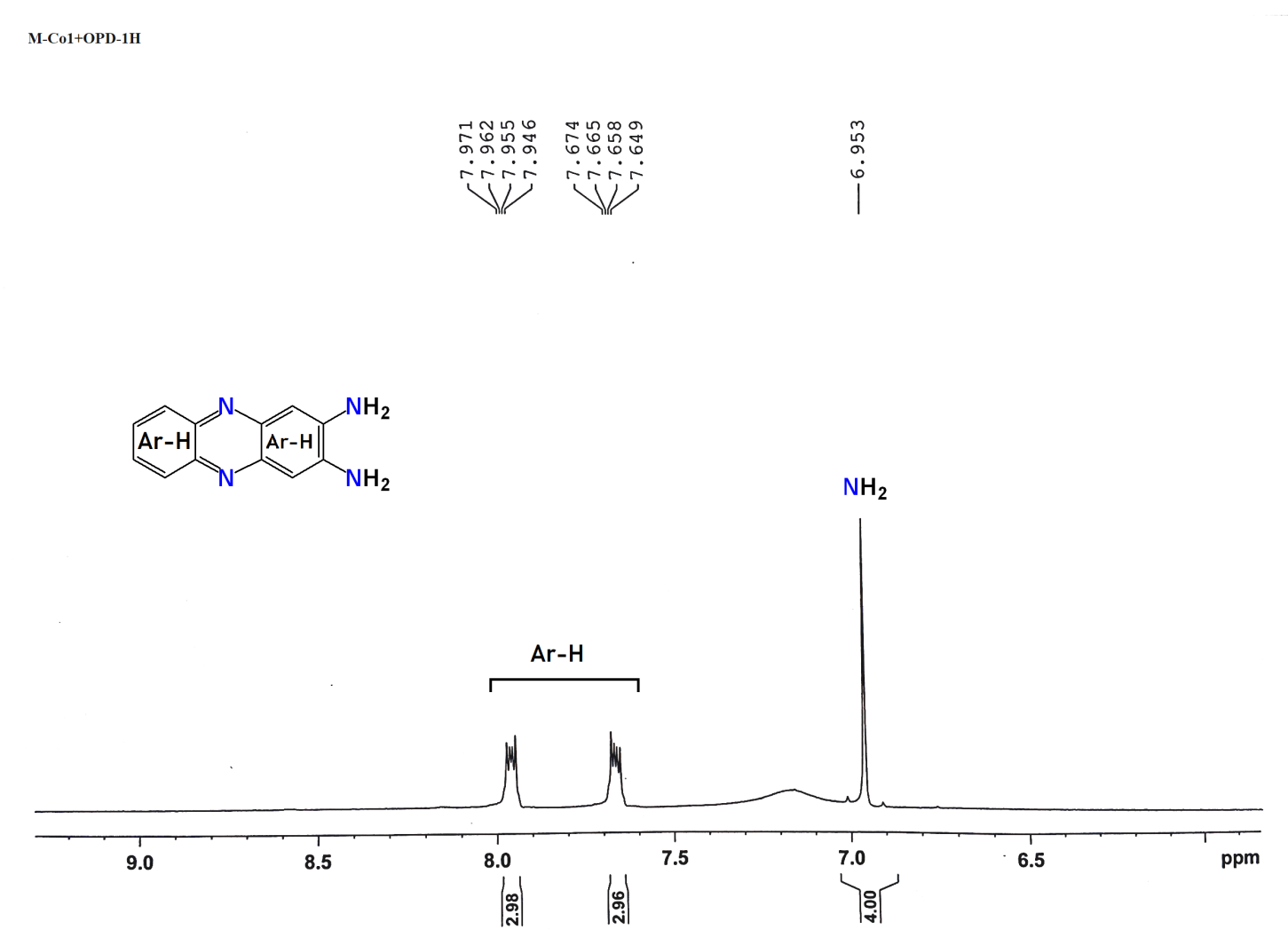
**

**Figure S3.** ^1^H NMR spectrum of DAPH^+^Cl^-^ in DMSO-d_6_.


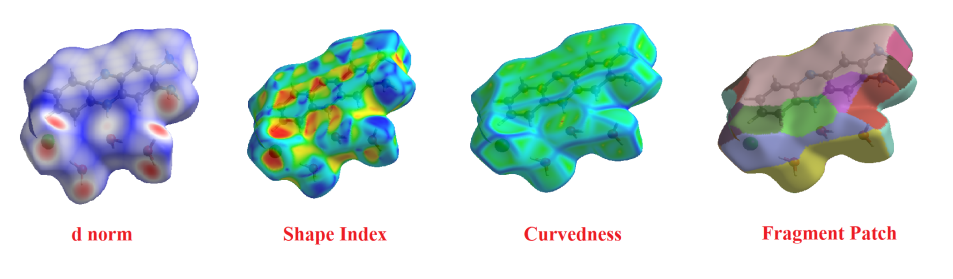


**Figure S4.** A view of Hirshfeld surfaces of DAPH^+^Cl^-^ mapped over d_norm_, shape index, curvedness and fragment patch


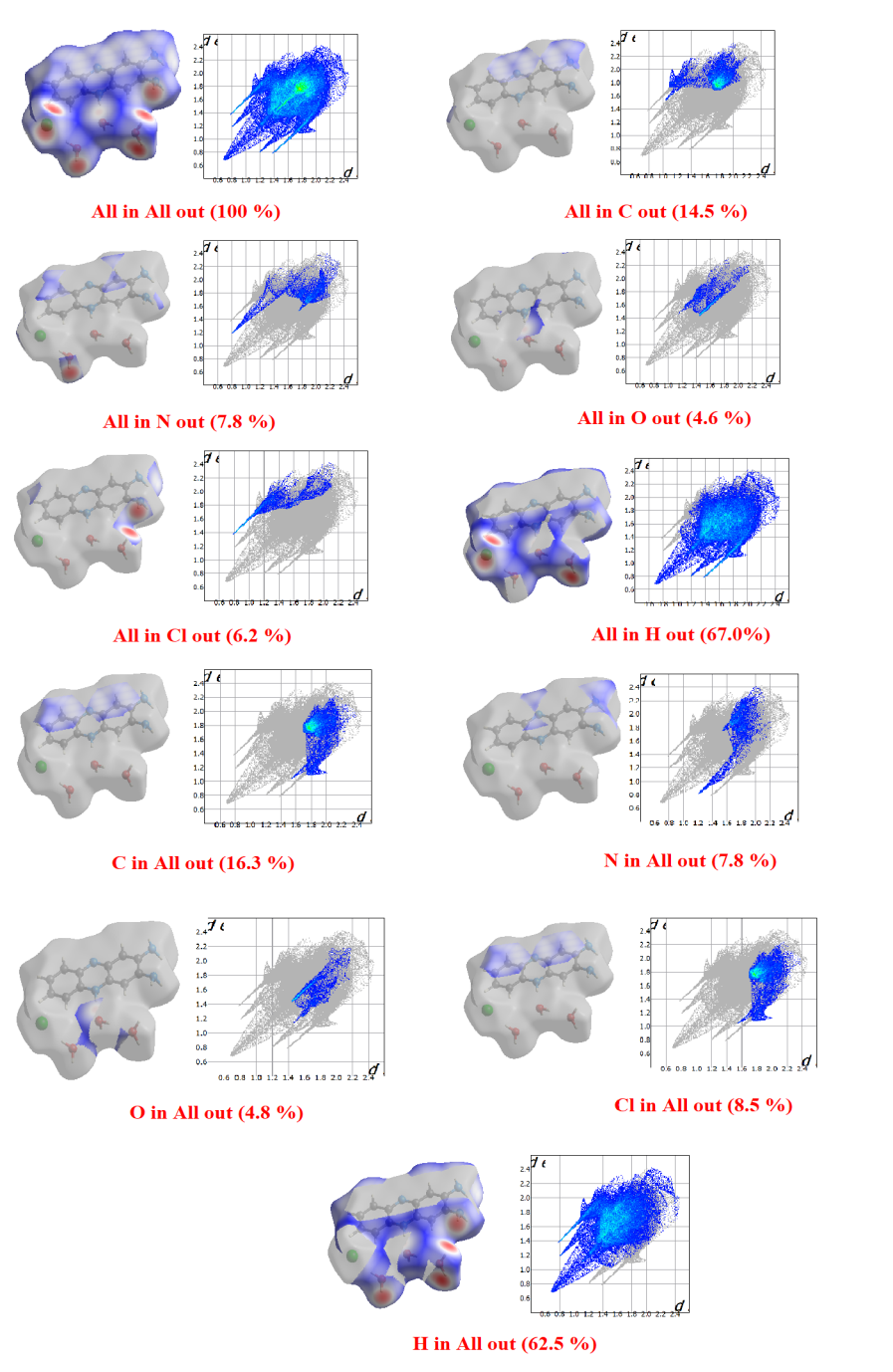


**Figure S5.** 2D fingerprint plots of DAPH^+^Cl^-^ with the contribution of interacting elements


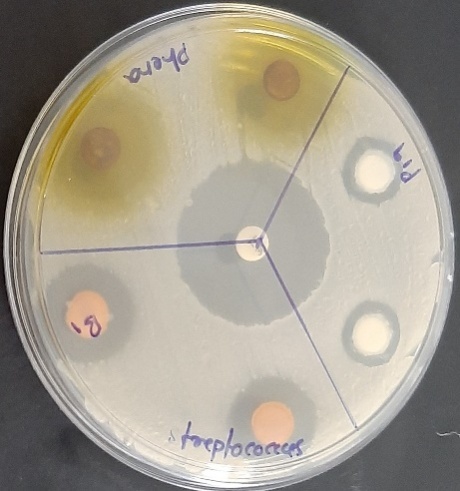

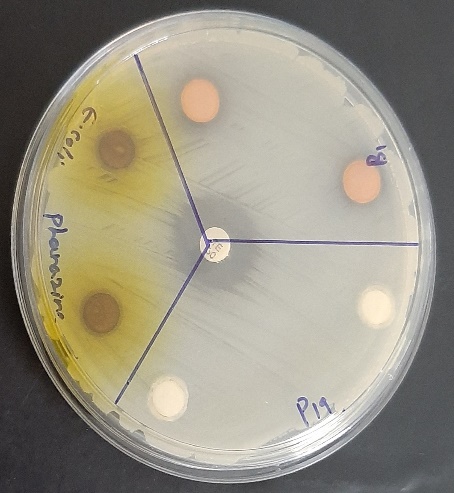

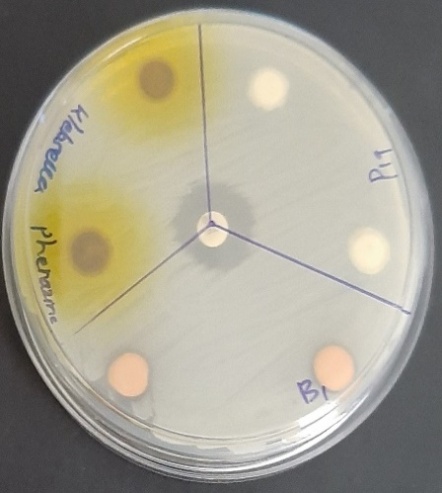


**Figure S6.** Antibacterial activity of a diindole-benzimidazole (B1), phenazinium chloride and tetracyclin against clinical microorganism


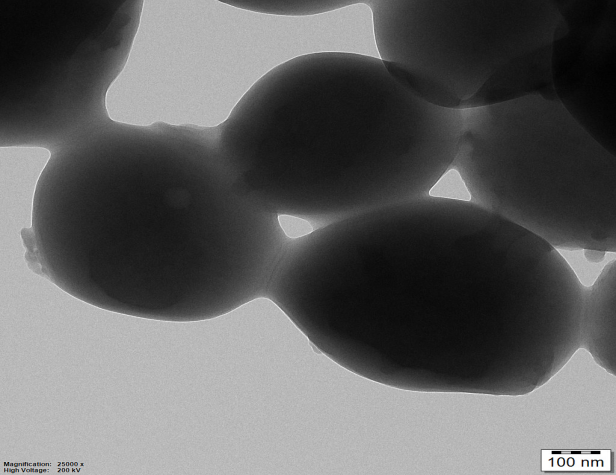

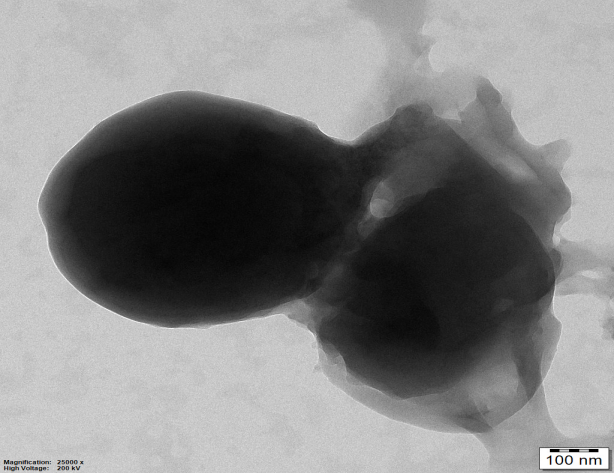


**Figure S7.** TEM observation of the phenazinum chloride-untreated control *Streptococcus pneumonia* (left); TEM image of phenazinum chloride-treated *Streptococcus pneumoniae*

**
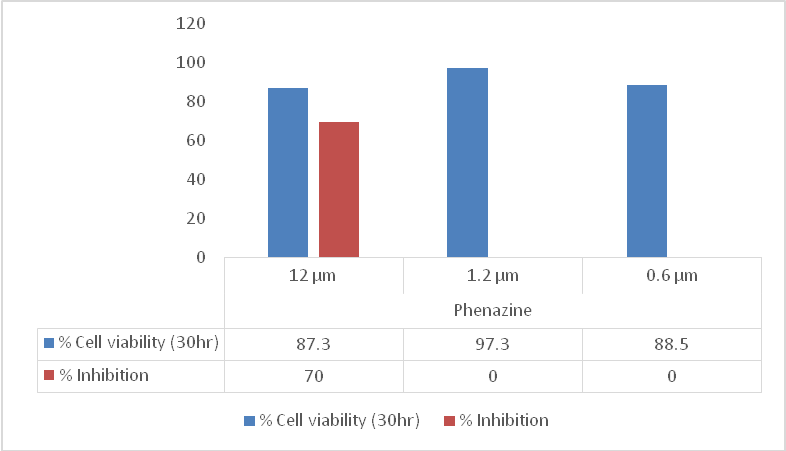
**

**Figure S8.** %Cell viability and viral inhibition activity of the phenazinium chloride on 1×10e4VeroE6 cells


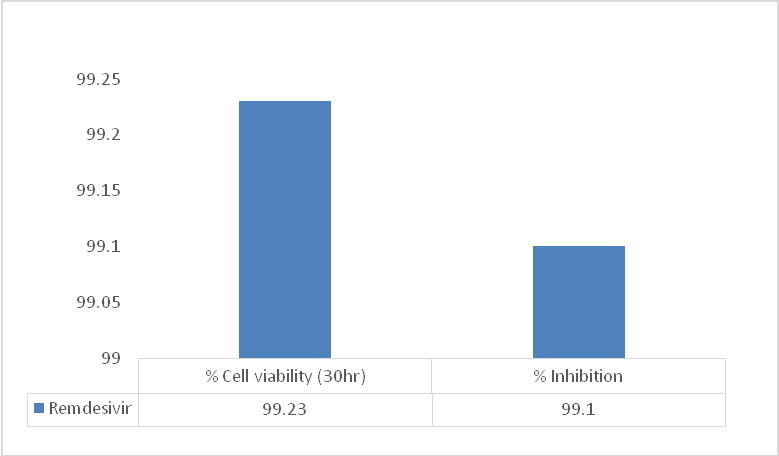


**Figure R1.** %Cell viability and viral inhibition activity of remdesivir and on 1×10e4VeroE6 cells

**Table S1.** Selected bond lengths (Å) and bond angles (°) for DAPH^+^Cl^-^

| Bond lengths | XRD | Bond lengths | XRD |
| --- | --- | --- | --- |
| N1-H1 | 0.8600 | N1-C1 | 1.369(3) |
| N2-C6 | 1.371(3) | N2-C7 | 1.337(3 |
| N1-C12 | 1.342(3) | N3 –C10 | 1.343(3) |
| C1 –C6 | 1.403(4) | C1 –C2 | 1.415(4) |
| C3 –C4 | 1.395(4) | C4 –C5 | 1.378(4) |
| C7 –C8 | 1.408(3) | C7 –C12 | 1.452(3) |
| C9 –C10 | 1.465(3) | C10 –C11 | 1.385(3) |
| C1-C6 | 1.408(3) | C7-C12 | 1.452(3) |
| N4 –C9 | 1.349(3) | C2 –C3 | 1.369(5) |
| C5 –C6 | 1.408(3) | C8 –C9 | 1.380(3) |
| C11 –C12 | 1.389(3) |  |  |
| Bond angles | XRD | Bond angles | XRD |
| C12-N1-H1 | 119.00 | C1-N1-H1 | 119.00 |
| C6-N2-C7 | 119.7(2) | N2-C7-C8 | 120.86(19) |
| C1-N1-C12 | 121.3(2) | N1-C1-C2 | 120.2(3) |
| N1-C1-C6 | 119.3(2) | C3-C4-C5 | 120.9(3) |
| C2-C3-C4 | 121.2(3) | C1-C6-C5 | 119.4(2) |
| N2-C6-C1 | 120.7(2) | C7-C8-C9 | 121.39(19) |
| C8-C7-C12 | 118.65(19) | N4-C9-C8 | 121.66(19) |
| C8-C9-C10 | 119.32(19) | N3-C10-C9 | 119.18(19) |
| N3-C10-C11 | 121.30(19) | C7-C12-C11 | 120.04(19) |
| N1-C12-C7 | 118.58(19) | N2-C7-C12 | 120.49(19) |
| C1-N1-C12 | 121.3(2) | N4-C9-C10 | 119.02(19) |
| N2-C6-C5 | 119.9(3) | C9-C10-C11 | 119.52(19) |
| C2-C1-C6 | 120.6(2) | C10-C11-C12 | 121.08(19) |
| C1-C2-C3 | 118.6(3) | N1-C12-C11 | 121.38(19) |
| C4-C5-C6 | 119.3(3) |  |  |

**Table S2**. Hydrogen bond parameters donor/acceptor scheme (Å,°) for DAPH^+^Cl^-^

| D-H…A | D-H | H…A | D…A | D-H…A | Symmetry code |
| --- | --- | --- | --- | --- | --- |
| N1-H1…O2 | 0.8600 | 1.8900 | 2.745(5) | 170 |  |
| O1-H1A…Cl1 | 0.8500 | 2.2400 | 3.087(3) | 171 |  |
| O1-H1B…N2 | 0.8500 | 2.1100 | 2.875(3) | 149 | x,-1+y,z |
| O2-H2A…O3 | 0.8500 | 2.0900 | 2.834(4) | 146 |  |
| N3-H3A…Cl1 | 0.8600 | 2.3800 | 3.228(2) | 170 | 1-x,-y,1-z |
| O3-H3C…N4 | 0.8500 | 2.3700 | 2.998(3) | 131 | x,-1+y,z |
| O3-H3D…Cl1 | 0.8500 | 2.3000 | 3.138(3) | 171 | 1-x,-y,1-z |
| N4-H4B…O3 | 0.8600 | 2.1700 | 2.998(3) | 161 | x,1+y,z |

**π…π** Interactions (Å,°) for DAPH^+^Cl^-^

| Cg...Cg | Cg-Cg distance | Symmetry code |
| --- | --- | --- |
| Cg(1)- Cg(3) | 3.5097 | -X,1-Y,1-Z |
| Cg(1)- Cg(3) | 3.5169 | 1-X,1-Y,1-Z |

**C-**H**…π** Interactions (Å, °) for DAPH^+^Cl^-^

| X-H...Cg | H..Cg | X...Cg | X-H...Cg(^o^) | Symmetry code |
| --- | --- | --- | --- | --- |
| N(4)-H(4A)....Cg(2) | 2.72 | 3.428(2) | 140 | -X,1-Y,1-Z |

**Table S3.** Interaction energies involved in the structure of DAPH^+^Cl^-^

|  | **N** | **Symop** | **R** | **Electron Density** | ***E´_ele_*** | ***E´_pol_*** | ***E´_disp_*** | ***E´_rep_*** | ***E´_tot_*** |
| --- | --- | --- | --- | --- | --- | --- | --- | --- | --- |
|  | 1 | *-* | 5.70 | HF/3-21G | 3.8 | -0.6 | -1.8 | 0.1 | 1.9 |
|  | 1 | *-x, -y, -z* | 11.49 | HF/3-21G | -2.2 | -0.3 | -5.6 | 1.5 | -6.2 |
|  | 1 | *-x, -y, -z* | 3.76 | HF/3-21G | -21.2 | -11.9 | -88.3 | 51.4 | -67.3 |
|  | 1 | *-* | 5.23 | HF/3-21G | 0.0 | nan | 0.0 | 0.0 | nan |
|  | 1 | *-* | 6.10 | HF/3-21G | 0.0 | nan | 0.0 | 0.0 | nan |
|  | 1 | *-x, -y, -z* | 10.46 | HF/3-21G | -1.0 | -0.2 | -6.1 | 1.2 | -5.6 |
|  | 1 | *-* | 6.74 | HF/3-21G | 0.0 | nan | 0.0 | 0.0 | nan |
|  | 1 | *-* | 7.92 | HF/3-21G | 2.5 | -0.7 | -5.2 | 0.9 | -1.9 |
|  | 1 | *-* | 6.70 | HF/3-21G | -2.2 | -0.3 | -5.6 | 1.5 | -6.2 |
|  | 1 | *-* | 6.26 | HF/3-21G | 0.8 | -0.2 | -0.8 | 0.0 | -0.0 |
|  | 1 | *-* | 5.78 | HF/3-21G | 2.9 | -0.7 | -2.2 | 0.2 | 0.8 |
|  | 1 | *-* | 5.39 | HF/3-21G | 0.5 | -0.0 | -0.3 | 0.0 | 0.1 |
|  | 2 | *x, y, z* | 12.86 | HF/3-21G | 0.0 | -0.3 | 0.0 | 0.0 | -0.2 |
|  | 1 | *-* | 7.08 | HF/3-21G | -21.2 | -11.9 | -88.3 | 51.4 | -67.3 |
|  | 1 | *-x, -y, -z* | 11.84 | HF/3-21G | -11.2 | -1.6 | -4.3 | 0.9 | -15.6 |
|  | 2 | *x, y, z* | 11.27 | HF/3-21G | 2.5 | -0.7 | -5.2 | 0.9 | -1.9 |
|  | 1 | *-* | 6.25 | HF/3-21G | -1.0 | -0.2 | -6.1 | 1.2 | -5.6 |
|  | 1 | *-* | 5.93 | HF/3-21G | 0.4 | -0.1 | -0.9 | 0.0 | -0.5 |
|  | 1 | *-* | 4.31 | HF/3-21G | -21.2 | -11.9 | -88.3 | 51.4 | -67.3 |
|  | 1 | *-* | 5.19 | HF/3-21G | 0.7 | -0.5 | -3.0 | 0.0 | -2.3 |
|  | 1 | *-x, -y, -z* | 3.71 | HF/3-21G | 21.5 | -12.6 | -70.2 | 30.0 | -25.2 |
|  | 1 | *-* | 5.58 | HF/3-21G | 2.3 | -1.2 | -3.7 | 1.1 | -0.9 |
|  | 1 | *-* | 4.14 | HF/3-21G | -21.2 | -11.9 | -88.3 | 51.4 | -67.3 |
|  | 1 | *-* | 6.85 | HF/3-21G | 0.7 | -0.5 | -3.0 | 0.0 | -2.3 |
|  | 1 | *-* | 7.91 | HF/3-21G | -0.6 | -1.0 | -4.1 | 1.2 | -4.0 |
|  | 1 | *-* | 6.12 | HF/3-21G | -26.0 | -8.1 | -4.7 | 18.5 | -20.9 |
|  | 1 | *-* | 5.77 | HF/3-21G | -0.9 | -0.0 | -0.4 | 0.0 | -1.3 |

Scale factors used to determine E_tot_: *k_ele_* = 1.019, *k_pol_* = 0.651, *k_disp_* = 0.901, *k_rep_*= 0.811 (Mackenzie et al., 2017)

**Table S4**. Hirshfeld surface plot and fingerprint plot of DAPH^+^Cl^-^

| Sr. no. | Inside | Outside | % surface area included |
| --- | --- | --- | --- |
|  | All | All | 100.0 |
|  | All | Cl | 6.2 |
|  | All | O | 4.6 |
|  | All | N | 7.8 |
|  | All | C | 14.5 |
|  | All | H | 67.0 |
|  | Cl | All | 8.5 |
|  | O | All | 4.8 |
|  | N | All | 7.8 |
|  | C | All | 16.3 |
|  | H | All | 62.5 |

**Table S5**. Antibacterial activity of DAPH^+^Cl^-^ and tetracycline against clinical microorganism

| S.NO | Test organism | Zone of Inhibition (mm) n=2 | | |
| --- | --- | --- | --- | --- |
|  |  |  | DAPH^+^Cl^-^ | Tetracycline |
| 1 | *Streptococcus* |  | 13 | 16 |
| 3 | *Escherichia coli* |  | 2 | 6 |
| 4 | *Klebsiella pneumoniae* |  | 2 | 5 |

(-) no zone of inhibition observed, standard antibiotic (Tetracycline)

**Table S6.** In vitro inhibition activity B1, phenazine and remdesivirviral on 1×10e4 VeroE6 cells

| Compound name | Concentration | % Cell viability (30hr) | % Inhibition |
| --- | --- | --- | --- |
| Remdesivir | 10 µm | 99.23 | 99.1 |
| Phenazine | 12 µm | 87.3 | 70 |
|  | 1.2 µm | 97.3 | 00.0 |
|  | 0.6 µm | 88.5 | 00.0 |

Note: The stock concentration of phenazine is 12 mg/mL

**Table S7.** Molecular docking interactions of DAPH^+^Cl^-^ and Mpro, nsp2 and nsp7-nps8

| Compound | Protein | Binding energy  (ΔG) | Type Interactions | Inhibition constant  (Ki) |
| --- | --- | --- | --- | --- |
| DAPH^+^Cl^-^ | Mpro | -6.66 kcal/mol | hydrogen bonding with Arg188, Thr190, Glu166; vdw interactions with Asp187, Met49, His 164, Pro168, Ala191, Leu167; **π**…**π** interaction with His 41 | 13.11µM |
| DAPH^+^Cl^-^ | nsp2 | -7.91 kcal/mol | hydrogen bonding with Cys72; vdw interactions with Asn108, Asn104, Met 75, Leu76; **C**…**π** interaction with Phe92, Leu95, Ile107 | 7.44 µM |
| DAPH^+^Cl^-^ | nsp7-nps8 | -7.12 kcal/mol | hydrogen bonding with Val11, Gln118; vdw interactions with Gln18, Trp29, Ser15, Val11, Ser15, Val83, Met87, Val12, Val12, Asn37; **π**…**π** interaction with Val33; **C**…**π** interaction with Leu14 | 8.61 µM |
| Remdesivir | PLpro | - | Four hydrogen bonding with Thr74, Asn128, Gln174 and Leu178 amino acid | - |
| Ivermectin | PLpro | - | hydrogen bonding with Thr74, Thr75, Asp76, Ala153 and His175 | - |

**Table S8.** The prediction of the ADME values for DAPH^+^Cl^-^

| **Physicochemical Properties** | |
| --- | --- |
| Formula | C12H10N4 |
| Molecular weight | 210.23 g/mol |
| Num. heavy atoms | 16 |
| Num. arom. heavy atoms | 14 |
| Fraction Csp3 | 0.00 |
| Num. rotatable bonds | 0 |
| Num. H-bond acceptors | 2 |
| Num. H-bond donors | 2 |
| Molar Refractivity | 65.85 |
| TPSA | 77.82 Å² |
| **Water solubility** | |
| Log *S* (ESOL) | -2.59 |
| Solubility | 5.39e-01 mg/ml; 2.56e-03 mol/l |
| Class | Soluble |
| Log *S* (Ali) | -2.50 |
| Solubility | 6.60e-01 mg/ml; 3.14e-03 mol/l |
| Class | Soluble |
| Log *S* (SILICOS-IT) | -4.29 |
| Solubility | 1.07e-02 mg/ml; 5.11e-05 mol/l |
| Class | Moderately soluble |
| **Liphophilicity** | |
| Log *P*_o/w_ (iLOGP) | 1.34 |
| Log *P*_o/w_ (XLOGP3) | 1.27 |
| Log *P*_o/w_ (WLOGP) | 1.96 |
| Log *P*_o/w_ (MLOGP) | 1.21 |
| Log *P*_o/w_ (SILICOS-IT) | 1.47 |
| Consensus Log *P*_o/w_ | 1.45 |
| **Pharmacokinetics** | |
| GI absorption | High |
| BBB permeant | No |
| P-gp substrate | Yes |
| CYP1A2 inhibitor | Yes |
| CYP2C19 inhibitor | No |
| CYP2C9 inhibitor | No |
| CYP2D6 inhibitor | Yes |
| CYP3A4 inhibitor | Yes |
| Log *K*_p_ (skin permeation) | -6.68 cm/s |
| **Druglikenes** | |
| Lipinski | Yes; 0 violation |
| Ghose | Yes |
| Veber | Yes |
| Egan | Yes |
| Muegge | Yes |
| Bioavailability Score | 0.55 |
