# Supplementary material for: A hydrated 2,3-diaminophenazinium chloride as a promising building block against SARS-CoV-2
Source: Sci Rep. 2021 Nov 30;11:23122. doi: 10.1038/s41598-021-02280-5 (PMC8633378; doi:10.1038/s41598-021-02280-5)
Supplement: Supplementary file 2 — Supplementary Information 2. [file 41598_2021_2280_MOESM2_ESM.docx]

**Supplementary data**

Supplementary crystallographic data are available free of charge from The Director, CCDC, 12 Union Road, Cambridge, CB2 1EZ, UK (fax: +44-1223-336033; E-mail: deposit@ccdc.cam.ac.uk or www: http://www.ccdc.cam.ac.uk) upon request, quoting deposition number CCDC 2090092. Experimental information such as FT-IR, UV-Vis, ^1^H & ^13^C NMR, Hirshfeld surface and fingerprint plot, bactericidal activity, selected bond distance and bond angles values, noncovalent interaction parameter and ADME properties etc are given here.
